# Supplementary material for: An interpretable approach to estimate the self-motion in fish-like robots using mode decomposition analysis
Source: Nat Commun. 2025 Apr 24;16:3887. doi: 10.1038/s41467-025-58880-6 (PMC12022055; doi:10.1038/s41467-025-58880-6)
Supplement: Supplementary file 1 — Supplementary Information [file 41467_2025_58880_MOESM1_ESM.pdf]

Supplementary Information for An Interpretable Approach to Estimate the Self-motion in Fish-like Robots Using Mode Decomposition Analysis:

Authors

Yufan Zhai[1], Xingwen Zheng[2,3], Li-Ming Chao[4,5,6], Shikun Li[1], Minglei Xiong[1], Yongxia Jia[7], Liang Li[4,5,6*], Guangming Xie[1,8*]

Affiliations

[1]State Key Laboratory for Turbulence and Complex Systems, Intelligent Biomimetic Design Lab, College of Engineering, Peking University, Beijing 100871, China

[2]Institute of Cyber-Systems and Control, Department of Control Science and Engineering, Zhejiang University, Hangzhou 310027, China

[3]State Key Laboratory of Ocean Sensing, Zhejiang University, Zhoushan, 316021, China

[4]Department of Collective Behaviour, Max Planck Institute of Animal Behavior, Konstanz 78464, Germany

[5]Centre for the Advanced Study of Collective Behaviour, University of Konstanz, Konstanz 78464, Germany

[6]Department of Biology, University of Konstanz, Konstanz 78464, Germany

[7]School of Aerospace Engineering, Tsinghua University, Beijing 100084, China

[8]Institute of Ocean Research, Peking University, Beijing 100871, China

*Corresponding authors: Liang Li (lli@ab.mpg.de), Guangming Xie (xiegming@pku.edu.cn)

Supplementary Note 1

Brief introduction of the research topic

This work aims to further solve the problem of self-state estimation for fish-like robots using ALLS from the perspective of mode decomposition (Supplementary Fig. 1). Three aspects of work have been conducted as follows.

Firstly, we use a typical mode decomposition method named Proper Orthogonal Decomposition (POD)[1] to analyze the experimental pressure data while the fish-like robot is swimming freely and the theoretical pressure data from the panel method[2]. The pressure variations on the surface can be decomposed into several modes strongly correlated with the motion states, such as the steady motion and oscillation, which is interpreted through Lighthill's theoretical pressure model[3]. Besides, based on the correlation between the coefficient and the velocity, we propose a method to estimate the velocity and trajectory of the fish-like robot. Furthermore, the energy distribution and mode 1 can be used to predict the optimal number and locations of sensors to reduce the redundancy of our ALLS design and improve the estimation.

Secondly, by comparing the trajectory estimation using different combinations of sensors, we validate the optimal prediction for the number and locations of sensors from POD. Compared with using all nine sensors, the trajectory estimation of the fish-like robot can be improved in the rectilinear and turning motions. In addition, flow visualization enabled by the hydrogen bubble technique is used to explain the different performances of the sensors from the perspective of flow structures.

Finally, we show that our method has strong generalizability:

1) The decomposition and estimation also work for the free-swimming fish-like robots under varying oscillation parameters.

2) The decomposition of three-dimensional pressure data of our boxfish model and eel-like model can also be interpreted by Lighthill's theoretical pressure model, suggesting our method could be extended

for other kinds of fish-like robots with different morphologies and swimming styles.

3) The estimation method based on mode decomposition is more robust in self-velocity estimation of the fish-like robot swimming in complex flows with vortices generated by a neighboring robot, compared with the regression method.

To sum up, with respect to the problem of self-state estimation for the fish-like robot using ALLS, POD makes a difference from three different aspects, providing a new perspective for interpreting the components of pressure data, improving the trajectory estimation by reducing the redundancy of ALLS, and showing generalizability under different situations. As a new approach, mode decomposition methods have the potential to further enhance the performance of ALLS in the future.

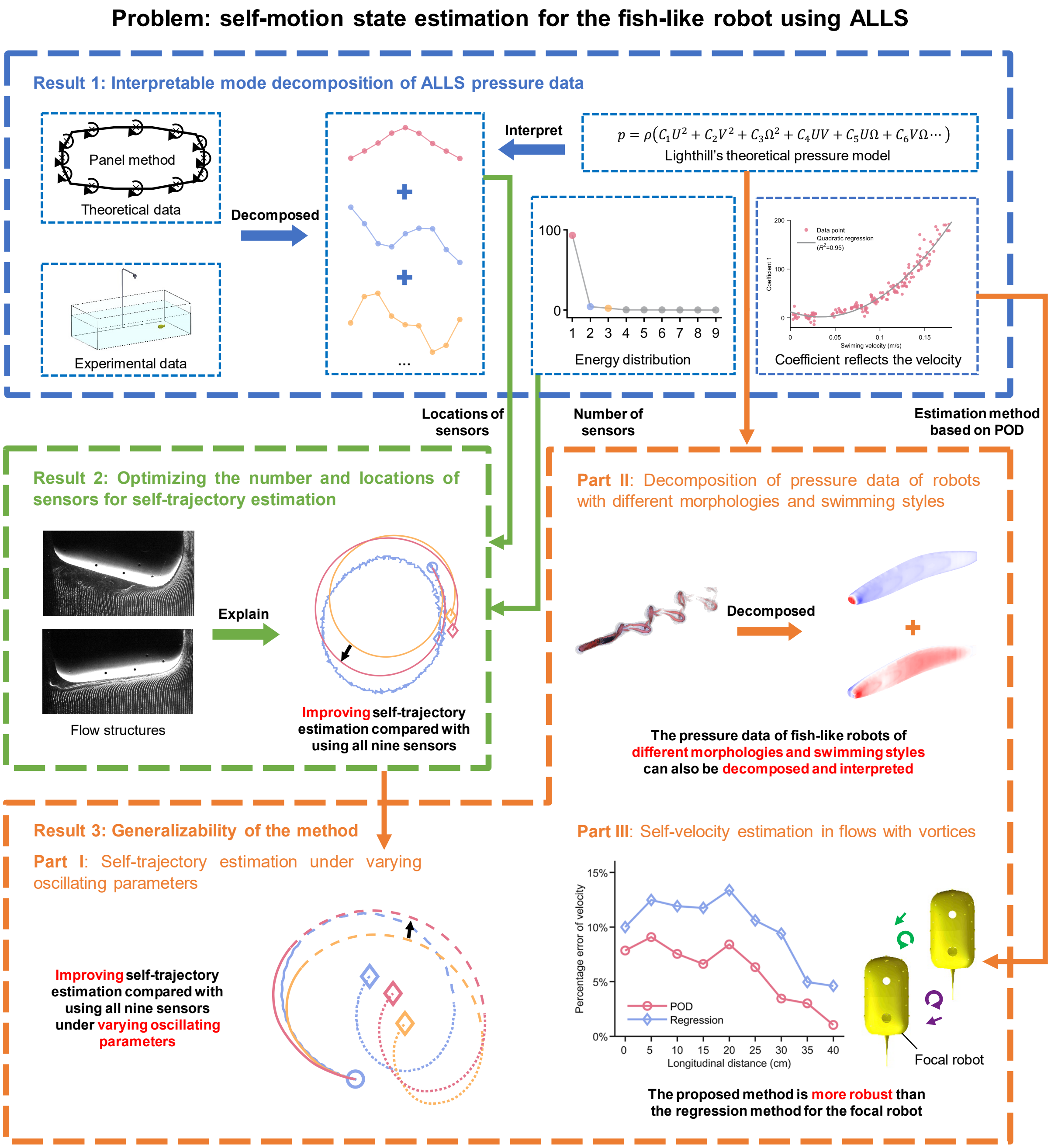

Supplementary Fig. 1 Graphical outline of the whole research topic. Our work is composed of three main parts presented in three different colored boxes.

Supplementary Note 2

Proper orthogonal decomposition (POD)

The POD algorithm based on singular value decomposition (SVD) is as follows. $\mathbf{A}(i,:)$ represents the ith row of matrix $\mathbf{A}$, and $\mathbf{A}(:,j)$ represents the jth column of matrix $\mathbf{A}$. $\mathbf{A}(i:k,j:m)$ represents the submatrix which is located on the ith to kth rows and jth to mth columns of matrix $\mathbf{A}$.

Algorithm 1: Proper orthogonal decomposition (POD)[1]

Input data $\mathbf{P} \in \mathbb{R}^{N\times M}$

  $[\mathbf{U}, \mathbf{S}, \mathbf{V}] = \mathrm{svd}(\mathbf{P})$

  Calculate energy of modes $E_i = S_{ii}^2$

  Sort modes according to E_i

  Select order of ROM r

  for $j = 1{:}r$

    $\mathbf{u}_j(\mathbf{x}) = \mathbf{U}(:,j)$

  for $j = 1{:}r$

    for $i = 1{:}M$

      $a_j(t_i) = \mathbf{P}(:,i) \cdot \mathbf{u}_j(\mathbf{x})$

Return $\mathbf{u}_j(\mathbf{x})$, $a_j(t_i)$

Supplementary Note 3

Theoretical model of hydrodynamic pressure on the surface

According to Lighthill's work[3], we consider a two-dimensional theoretical model that describes the hydrodynamic pressure variations on the surface of a swimming fish-like robot based on potential flow theory. It is assumed here that the flow remains irrotational and the boundary layer effect is absent. It is also worth mentioning that we mainly focus on the ALLS data, namely the pressure on the surface. Therefore, we ignore the tail oscillation in the model given that the vortices caused by the tail propagates backward and the impact on the surface pressure is insignificant[3,4].

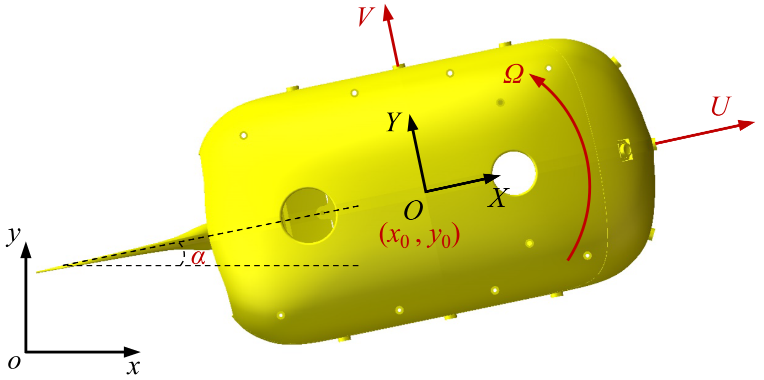

Supplementary Fig. 2 Kinematic diagram for two-dimensional swimming. oxy denotes the global inertial coordinate frame. OXY denotes the body-fixed coordinate frame. (x_0, y_0) denotes the global coordinates of the fish-like robot mass center. α denotes the absolute yaw angle. U, V denote the velocity components in OXY. Ω denotes the angular velocity, which is positive in counterclockwise direction.

In the two-dimensional model, the motion of the fish-like robot is shown in Supplementary Fig. 2. Based on Lighthill's theoretical pressure model[3], the velocity potential function can be decomposed into a combination of basic potential functions caused by three motion states

$$\varphi(t) = U(t)\varphi_f(X,Y) + V(t)\varphi_l(X,Y) + \Omega(t)\varphi_r(X,Y) \tag{S1}$$

where U, V, Ω are functions of time t and represent the real-time velocity and angular velocity. φ_f, φ_l, and φ_r are three basic functions that represent the velocity potential functions of the flow field caused by unit

forward velocity, unit lateral velocity, and unit rotational velocity, respectively. Thus, the independent variables of the functions are the relative coordinates X and Y in the body-fixed coordinate frame. There exists a transformation between global coordinates and relative coordinates

$$\begin{aligned} X &= (x - x_0)\cos\alpha + (y - y_0)\sin\alpha \\ Y &= (y - y_0)\cos\alpha - (x - x_0)\sin\alpha \\ U &= \dot{x}_0\cos\alpha + \dot{y}_0\sin\alpha \\ V &= \dot{y}_0\cos\alpha - \dot{x}_0\sin\alpha \end{aligned} \tag{S2}$$

Based on unsteady Bernoulli's equation

$$p(t) = -\rho\frac{\partial\varphi}{\partial t} - \frac{1}{2}\rho\|\nabla\varphi\|^2 \tag{S3}$$

where $p(t)$ represents the real-time hydrodynamic pressure relative to the pressure value in still water. ρ represents the density of water. It can be obtained that

$$\begin{aligned} \frac{p}{\rho} &= -\frac{\partial\varphi}{\partial t} - \frac{1}{2}\|\nabla\varphi\|^2 \\ &= C_1U^2 + C_2V^2 + C_3\Omega^2 + C_4UV + C_5U\Omega + C_6V\Omega + C_7\frac{dU}{dt} + C_8\frac{dV}{dt} + C_9\frac{d\Omega}{dt} \end{aligned} \tag{S4}$$

where

$$\begin{aligned} C_1 &= \frac{\partial\varphi_f}{\partial X} - \frac{1}{2}\left\|\nabla\varphi_f\right\|^2 \\ C_2 &= \frac{\partial\varphi_l}{\partial Y} - \frac{1}{2}\|\nabla\varphi_l\|^2 \\ C_3 &= -Y\frac{\partial\varphi_r}{\partial X} + X\frac{\partial\varphi_r}{\partial Y} - \frac{1}{2}\|\nabla\varphi_r\|^2 \\ C_4 &= \frac{\partial\varphi_l}{\partial X} + \frac{\partial\varphi_f}{\partial Y} - \nabla\varphi_f\cdot\nabla\varphi_l \\ C_5 &= \frac{\partial\varphi_r}{\partial X} - Y\frac{\partial\varphi_f}{\partial X} + X\frac{\partial\varphi_f}{\partial Y} - \nabla\varphi_f\cdot\nabla\varphi_r \\ C_6 &= \frac{\partial\varphi_r}{\partial Y} - Y\frac{\partial\varphi_l}{\partial X} + X\frac{\partial\varphi_l}{\partial Y} - \nabla\varphi_l\cdot\nabla\varphi_r \\ C_7 &= -\varphi_f \\ C_8 &= -\varphi_l \\ C_9 &= -\varphi_r \end{aligned} \tag{S5}$$

How to calculate C_i in Lighthill's pressure model

The panel method[2] based on the same assumption of the potential flow can calculate the pressure with prior knowledge of the motion of the fish-like robot. C_i in Eq. (S4) can be calculated by setting the velocity components to specific values. For example, by setting the forward velocity U of the fish-like robot to a constant and the velocity components V, Ω to zero, the pressure on the surface, which equals C_1U^2 according to Eq. (S4), can be used for comparison with POD modes (dashed lines in Fig. 2). $C_3\Omega^2$ can also be calculated by setting that the robot only has a constant angular velocity. Since that the movement of the fish-like robot is relatively stable, the acceleration terms in Eq. (S4) are less significant. And the terms with V are also less significant because our fish-like robot swims forward nearly without lateral velocity. Therefore, we only calculate the pressure caused by U and Ω for comparison with the dominant modes in POD.

Differences between the theoretical data from the panel method and experimental data

There exist some quantitative differences between the experimental data and the theoretical data or Lighthill's pressure model because the potential flow theory[2], as a simplified approach, offers a foundational understanding of the hydrodynamic pressure around a swimming object but does not account for many real-world complexities. These include boundary layer effects, wake vortices generated by the tail, and fluid-structure coupling interactions, all of which play significant roles in real environments. In terms of mode 1, theoretical data is different from experimental data at sensors L_2, R_2 in the rectilinear motion (Fig. 2**b**) and L_1, L_2 in the turning motion (Fig. 3**b**). It may be due to the large curvature of the shell shape, resulting in flow separation, vortices, and complicated pressure variations, which are shown in the flow visualization (Figs. 4**a**, 5**a**, and 5**b**). In addition, in theoretical data, the mode with the highest energy proportion corresponds to oscillation rather than steady motion (Figs. 2**b** and 3**b**). This is also because that the theoretical method does not consider the influence of vortices on pressure variations and thus obtains that the pressure generated by oscillation is more significant. In the experimental data, the complex pressure variations caused by vortices could be eliminated by POD, so the dominant mode corresponds to steady motion and can be used for estimation. Furthermore, in theoretical data, the dominant mode corresponding

to oscillation can be eliminated by taking the average value over time. So 'theoretical sensitivity' can be calculated by the mode corresponding to steady motion even it is not dominant.

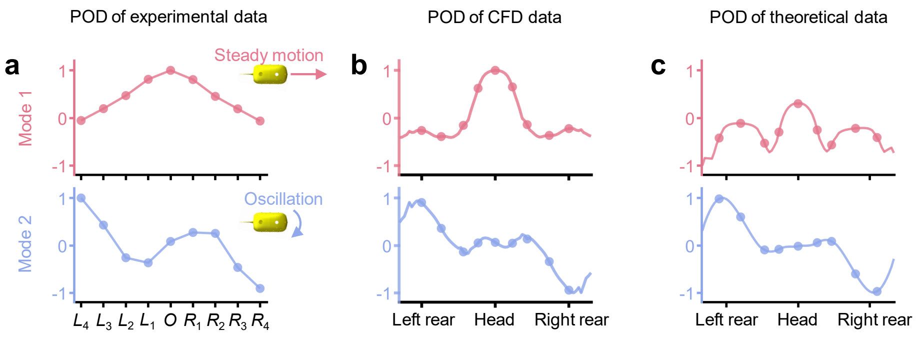

Supplementary Fig. 3 The decomposed modes 1 and 2 of experimental, CFD simulation and theoretical pressure data on the two-dimensional plane. **a**, Experimental data. **b**, CFD simulation data. **c**, Theoretical data.

Another reason may be that the theoretical data and Lighthill's pressure model are both 2D, which is different from the 3D fish body. With further experiments based on CFD simulation of a 3D free-swimming fish-like robot which consider the viscosity, we do find the discrepancies between the experiments and 3D simulations reduce, as shown in Supplementary Fig. 3. This indicates that the 3D morphology and viscosity, which are largely ignored in Lighthill's pressure model and the panel method, could be the main reasons causing such discrepancies.

Supplementary Note 4

How to correlate the coefficients in POD to the motion states in Lighthill's pressure model

Coefficient 1 primarily reflects the variations in velocity U. Although dU/dt could theoretically influence the hydrodynamic pressure, its contribution is less significant in this problem, as the movement of the fish-like robot is relatively stable without sudden acceleration or deceleration. As shown in Supplementary Figs. 15, 17, the decomposition results of the experimental data under varying oscillation parameters indicate that coefficient 1 has consistently remained positive, even during the deceleration phase ($dU/dt < 0$). The pressure distribution caused by forward velocity U and acceleration dU/dt should have a maximum value at the head, as the stagnation point, and decrease toward the sides. Therefore, the acceleration components could be coupled into mode 1 and coefficient 1. To conclude, coefficient 1 primarily reflects the variation in velocity U, while also incorporating some acceleration information. But our main focus is on estimating the swimming velocity and trajectory of the fish-like robot, so we have not placed much emphasis on the acceleration information. In future research, if we aim to use mode decomposition methods to explore the perception and control of the fish-like robot under abrupt disturbances that generate large accelerations, the acceleration terms dU/dt will become more important. Regarding whether coefficient 1 is correlated with U or U^2, our goal is to intuitively present the qualitative conclusion that coefficient 1 reflects the variation in velocity, increasing or decreasing simultaneously. For the quantitative relationship between the two, we have established a quadratic model $\overline{\mathrm{coef}_1(t)} = aU^2 + bU + c$, which includes both linear and quadratic terms, for subsequent estimation.

Coefficient 2 reflects variations in rotational angular velocity Ω, rather than Ω^2, because coefficient 2 fluctuates between positive and negative values and exhibits the same frequency as Ω. If coefficient 2 were related to Ω^2, it would always be positive and have a frequency twice that of the angular velocity Ω, which is not observed. Additionally, there may be a phase difference between coefficient 2 and the angular velocity, as $d\Omega/dt$ could cause the pressure distribution on the surface similar to mode 2. The amplitude of angular

acceleration is equal to the amplitude of angular velocity multiplied by $2\pi f$, so these two terms may couple in mode 2 and coefficient 2, resulting in a slight phase difference.

Coefficient 3 is related to $U\Omega$, as observed by comparing mode 3 with coefficient C_5, which corresponds to $U\Omega$ in Lighthill's pressure model. However, this conclusion requires further investigation, as mode 3, with its relatively low energy proportion, is not dominant and may be influenced by other factors. The hydrodynamic pressure caused by angular acceleration and lateral velocity could also exhibit the same antisymmetry as mode 3, which may explain the differences between coefficient 3 and the variation in $U\Omega$. For reference, we choose $U\Omega$ as the label on the right panel. In this study, our primary focus is on mode 1 and coefficient 1 for velocity estimation. In future work, we plan to further investigate and interpret the remaining modes, exploring how they can be used to predict additional states for the fish-like robot, such as angular velocity and acceleration.

Supplementary Note 5

Quadratic relationships between POD coefficients and swimming velocity

It is evident that quadratic relationships exist between the average value of the coefficient of mode 1 and the swimming velocity in both rectilinear and turning motions (Supplementary Fig. 4). The quadratic relationship observed in the turning motion (Supplementary Fig. 4**b**) is insignificant because the velocity ranges from 0.03 to 0.09 m/s due to the limitation of the experimental platform. In the rectilinear motion, the quadratic relationship is evident when the velocity is larger than 0.1 m/s (Supplementary Fig. 4**a**).

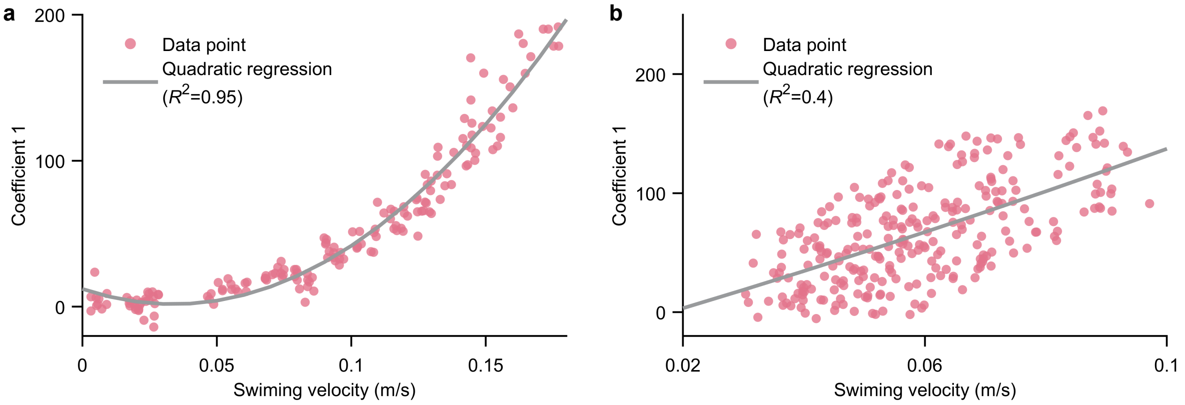

Supplementary Fig. 4 Quadratic relationships between the coefficient of mode 1 using all nine sensors and the swimming velocity. **a**, Rectilinear motion. **b**, Turning motion.

Supplementary Note 6

Decomposition results of more cases under different oscillation parameters

We also provide the decomposition results of the pressure data for rectilinear and turning motions under different oscillation frequencies, amplitudes, and offsets, which are shown in Supplementary Figs. 5, 6. The pressure data in different cases can be decomposed into several interpretable modes and coefficients, as described in our manuscript. The first three modes account for almost all of the energy. For the rectilinear motion, modes 1 exhibit left-right symmetry and reaches the highest value at the head, and coefficients 1 fluctuate around a positive value because the fish-like robot has reached a stable velocity in the selected segments. Modes 2 exhibits antisymmetry, and coefficients 2 reflect the variation in angular velocity. Modes 3 and coefficients 3 may reflect the coupling terms and other terms in Lighthill's pressure model. For the turning motion, modes 1 is asymmetrical since the fish-like robot is turning right. Overall, the decomposition results may not be affected by different oscillation parameters within the normal operating range of our fish-like robot.

There are also some differences among different cases which can also be interpreted. Firstly, the energy proportion of the first mode, which is related to the steady motion, depends on the swimming velocity. A larger swimming velocity causes a larger energy proportion of the first mode. Besides, the variation range of coefficient 2, which is related to the oscillation, depends on the oscillation amplitude of the tail and body. A larger oscillation amplitude causes a larger variation range of coefficient 2. These are because larger swimming velocities and oscillation amplitudes generate stronger hydrodynamic pressure signals around the surface of the fish-like robot.

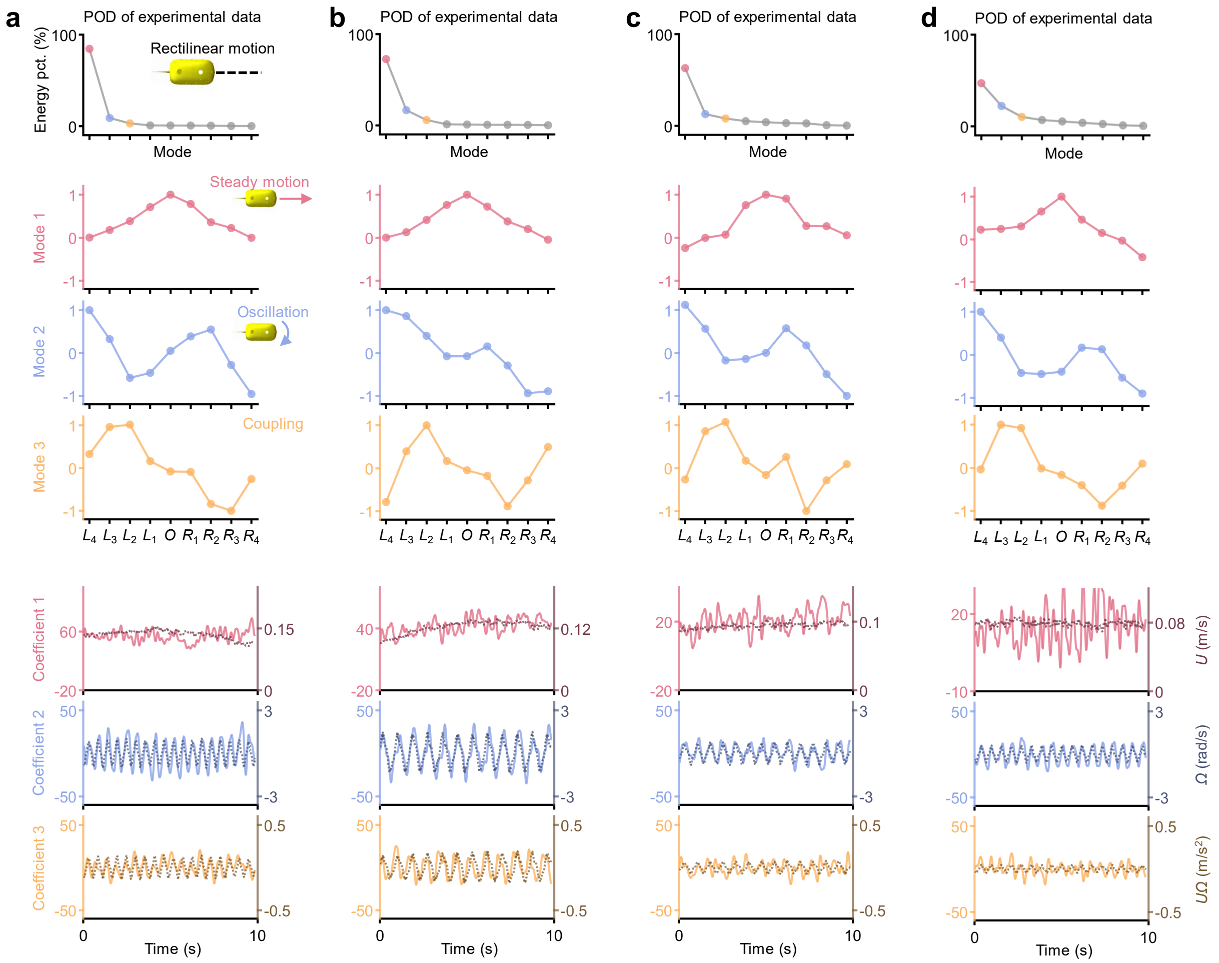

Supplementary Fig. 5 Mode decomposition (POD) results of the experimental hydrodynamic pressure data of more cases in the rectilinear motion. The points, lines, and colors in this figure follow the same legend as Fig. 2. The first three modes almost occupy all the energy. Mode 1 and coefficient 1 are related to the steady motion. Mode 2 and coefficient 2 are related to the oscillation. Mode 3 and coefficient 3 are related to other coupling motions. **a**, Frequency = 2 Hz, amplitude = 20°, and offset = 0°. **b**, Frequency = 1.2 Hz, amplitude = 30°, and offset = 0°. **c**, Frequency = 1.4 Hz, amplitude = 15°, and offset = 0°. **d**, Frequency = 1.8 Hz, amplitude = 10°, and offset = 0°.

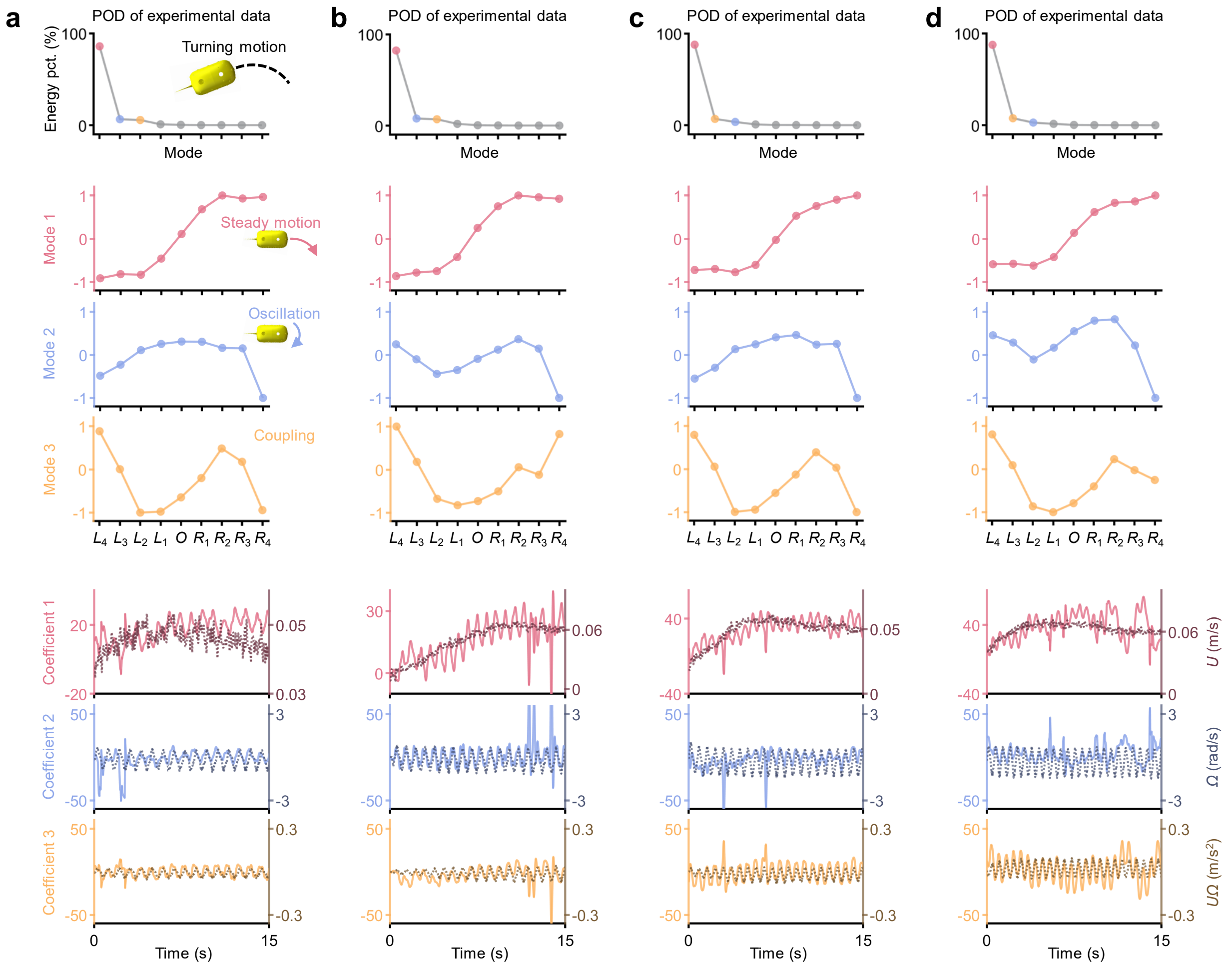

Supplementary Fig. 6 Mode decomposition (POD) results of the experimental hydrodynamic pressure data of more cases in the turning motion. The points, lines, and colors in this figure follow the same legend as Fig. 3. The first three modes almost occupy all the energy. Mode 1 and coefficient 1 are related to the steady motion. Mode 2 and coefficient 2 are related to the oscillation. Mode 3 and coefficient 3 are related to other coupling motions. **a**, Frequency = 1.1 Hz, amplitude = 20°, and offset = 30°. **b**, Frequency = 1.3 Hz, amplitude = 20°, and offset = 25°. **c**, Frequency = 1.5 Hz, amplitude = 20°, and offset = 35°. **d**, Frequency = 1.7 Hz, amplitude = 20°, and offset = 30°.

Supplementary Note 7

‘Sensitivity’ from POD mode 1

Mode 1 from POD is similar to the actual pressure variations when velocity components go through possible values (Supplementary Fig. 7). As for the rectilinear motion, V and Ω equal 0 on average. Only U ranges from 0 to 0.2 m/s. As for the turning motion, the ranges of U, V, Ω are 0 to 0.2 m/s, 0 to 0.05 m/s, and 0 to 0.5 rad/s, respectively. In traversal, the ‘theoretical sensitivity’ is calculated by the panel method[2]. The ‘experimental sensitivity’ directly comes from the experimental data.

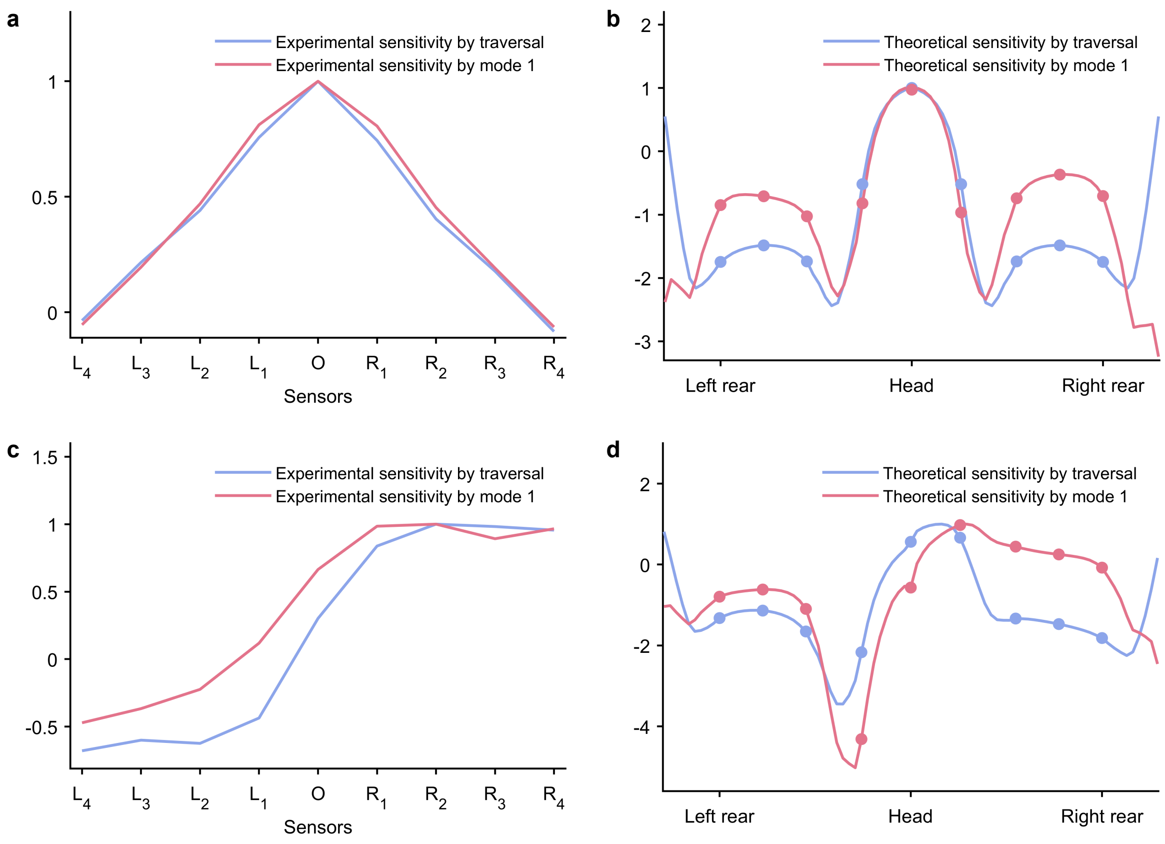

Supplementary Fig. 7 Comparison between ‘experimental sensitivity’, ‘theoretical sensitivity’ from POD mode 1 and traversal. **a**, ‘Experimental sensitivity’ in the rectilinear motion. **b**, ‘Theoretical sensitivity’ in the rectilinear motion. **c**, ‘Experimental sensitivity’ in the turning motion. **d**, ‘Theoretical sensitivity’ in the turning motion.

Improvement in trajectory estimation by using the optimal combination of sensors

The trajectory estimation can be improved by using the optimal combination of sensors compared with using all nine sensors for both methods. In the rectilinear motion, the improvement is not apparent because the estimation using all nine sensors is satisfactory enough (Supplementary Fig. 8). In the turning motion, the improvement is apparent (Supplementary Fig. 9).

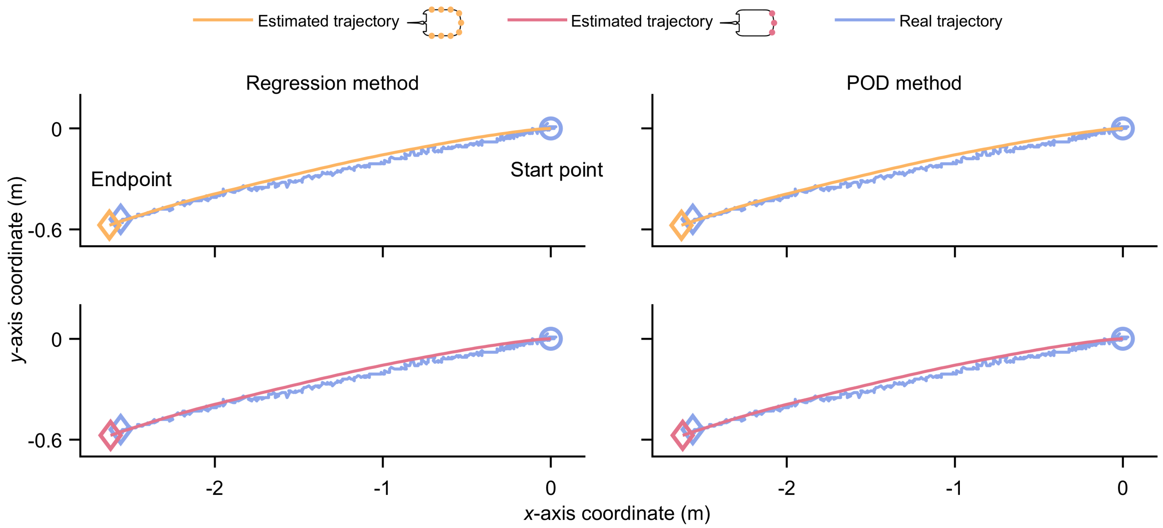

Supplementary Fig. 8 Trajectory estimation by using all nine sensors and the optimal combination of sensors in the rectilinear motion. Blue circles, lines, and diamonds represent the start point, real trajectory, and real endpoint. Orange lines and diamonds represent the estimated trajectory and endpoint using all nine sensors. Red lines and diamonds represent the estimated trajectory and endpoint using the optimal combination of sensors.

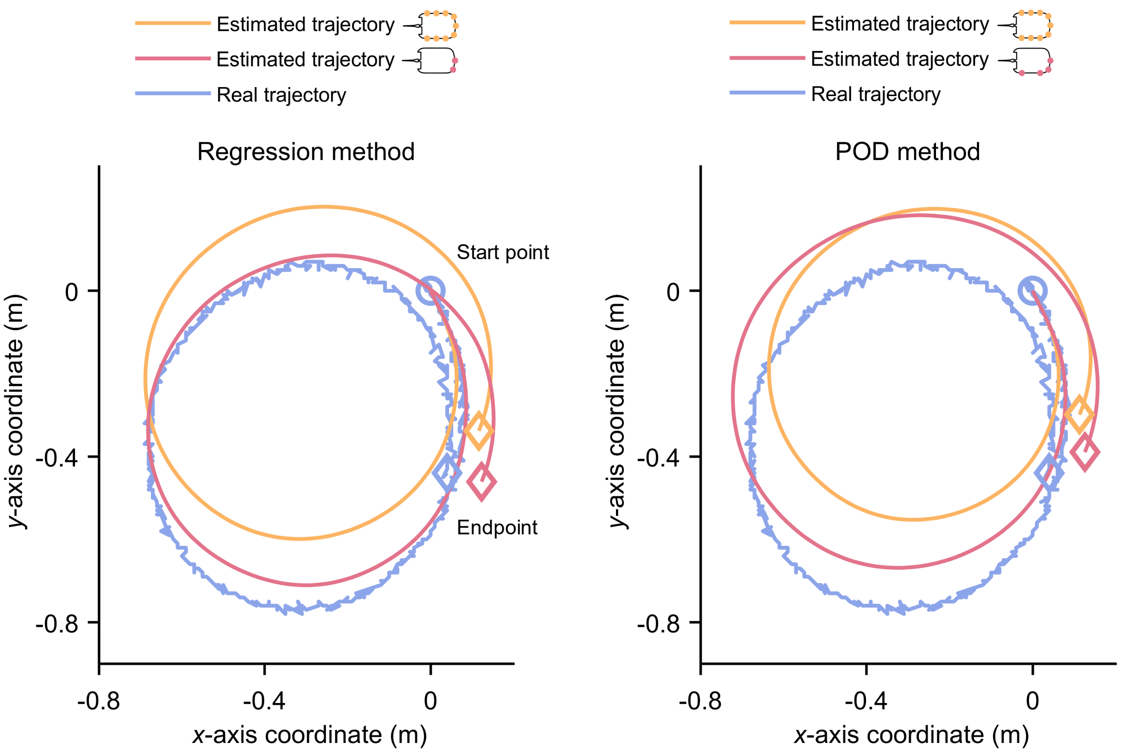

Supplementary Fig. 9 Trajectory estimation by using all nine sensors and the optimal combination of sensors in the turning motion. Blue circles, lines, and diamonds represent the start point, real trajectory, and real endpoint. Orange lines and diamonds represent the estimated trajectory and endpoint using all nine sensors. Red lines and diamonds represent the estimated trajectory and endpoint using the optimal combination of sensors.

Evaluating sensor locations

Supplementary Fig. 10 shows the amplitude of the dominant frequency of the pressure data, calculated by Fast Fourier Transform (FFT), for each sensor in the turning motion over a long time. Although data from sensors L_4, L_3, L_2, L_1 have larger amplitudes, they are not potentially optimal locations. It may be because vortices have a tremendous negative impact on the pressure signals and even cover the effective components used for trajectory estimation. For the sensors O, R_1, R_2, R_3, R_4 in the laminar flow area with lower noise levels, a larger amplitude means a larger signal-to-noise ratio, which will lose less information during signal processing. This can explain why R_4 is included in the optimal combination of sensors although it may be affected by vortices near the rear part.

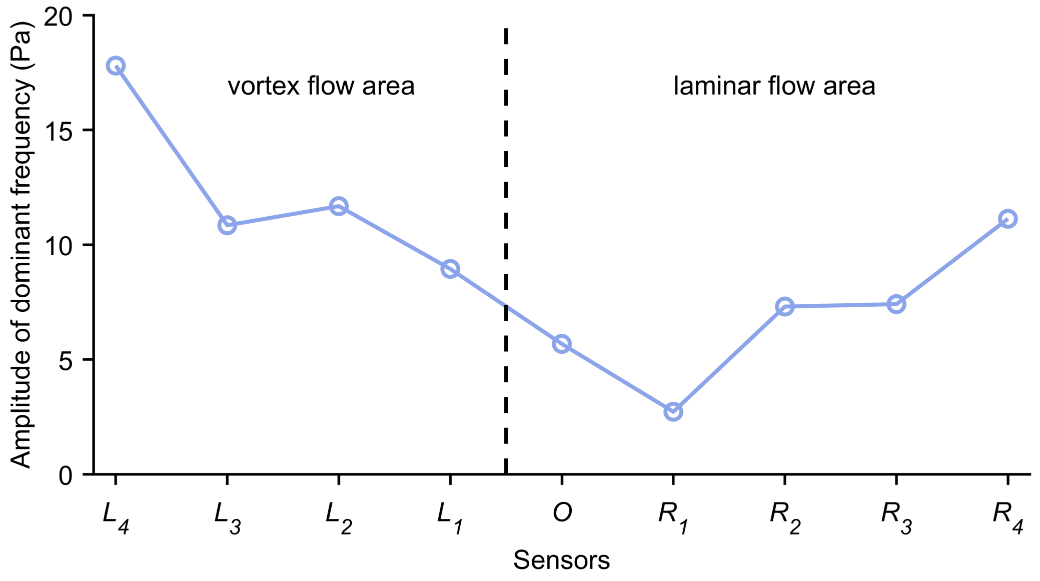

Supplementary Fig. 10 Amplitude of the dominant frequency of each sensor in the turning motion by FFT.

Supplementary Note 8

Trajectory estimation of more cases under different oscillation parameters

We also provide trajectory estimation results of more cases for rectilinear and turning motions under different oscillation frequencies, amplitudes, and offsets, which are shown in Supplementary Figs. 11-14. Overall, the performance of the regression and POD methods for estimating the self-trajectory of the fish-like robot is satisfactory. The optimal combinations of the pressure sensors are consistent with the conclusion in our manuscript. For the rectilinear motion, the minimum error is reached by using three sensors L_1, O, R_1 for both methods, which is consistent with the prediction based on POD. The more sensors that are used, the higher the error.

For the turning motion, the optimal combinations of sensors mostly appear on the right front part of the body. The optimal number of sensors could be three, four, or even five, which appears to be larger than the three suggested by POD. Firstly, regardless of whether three, four, or five sensors on the right front part are used, the estimation error is lower than using all nine sensors. The only optimal combination may differ due to experimental fluctuations and sensor noise. Some suboptimal combinations also perform better than using all nine sensors. Besides, the optimal number is predicted by the energy proportion of modes in POD. The question of how many modes are needed to capture almost all of the information depends on the system's characteristics and the level of noise. The total energy of the first few modes should be larger than the threshold which could be 90%, 95% or 99%. It may lead to fluctuations in the number of selected modes, which can explain why the optimal number of sensors is not a fixed value in this problem.

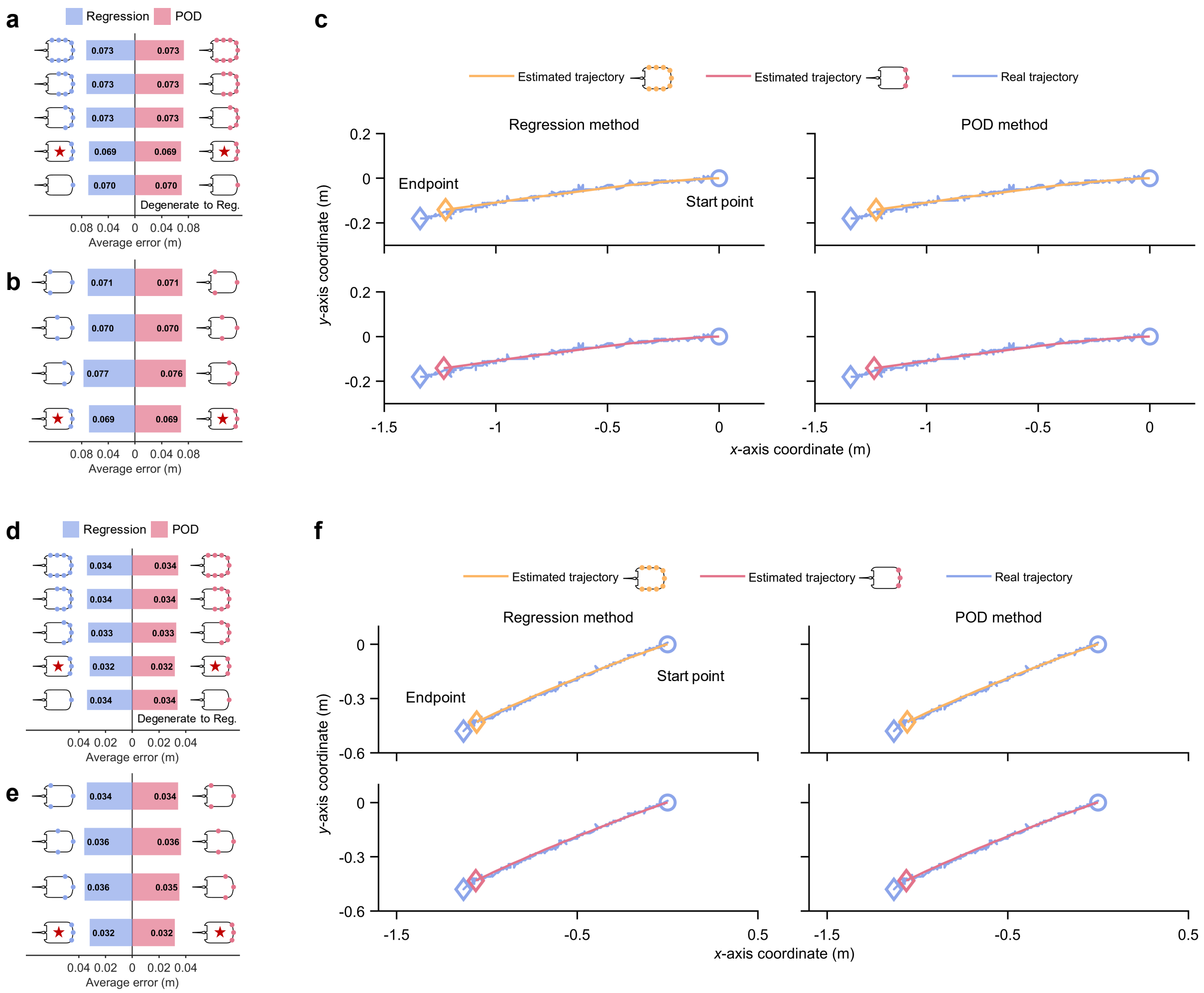

Supplementary Fig. 11 Trajectory estimation of more cases in the rectilinear motion. The points, lines, and colors in this figure follow the same legend as Fig. 4 and Fig. 6. The minimum error is reached by using L_1, O, R_1 for both methods. **a b c**, Frequency = 2 Hz, amplitude = 20°, and offset = 0°. **d e f**, Frequency = 1.2 Hz, amplitude = 30°, and offset = 0°.

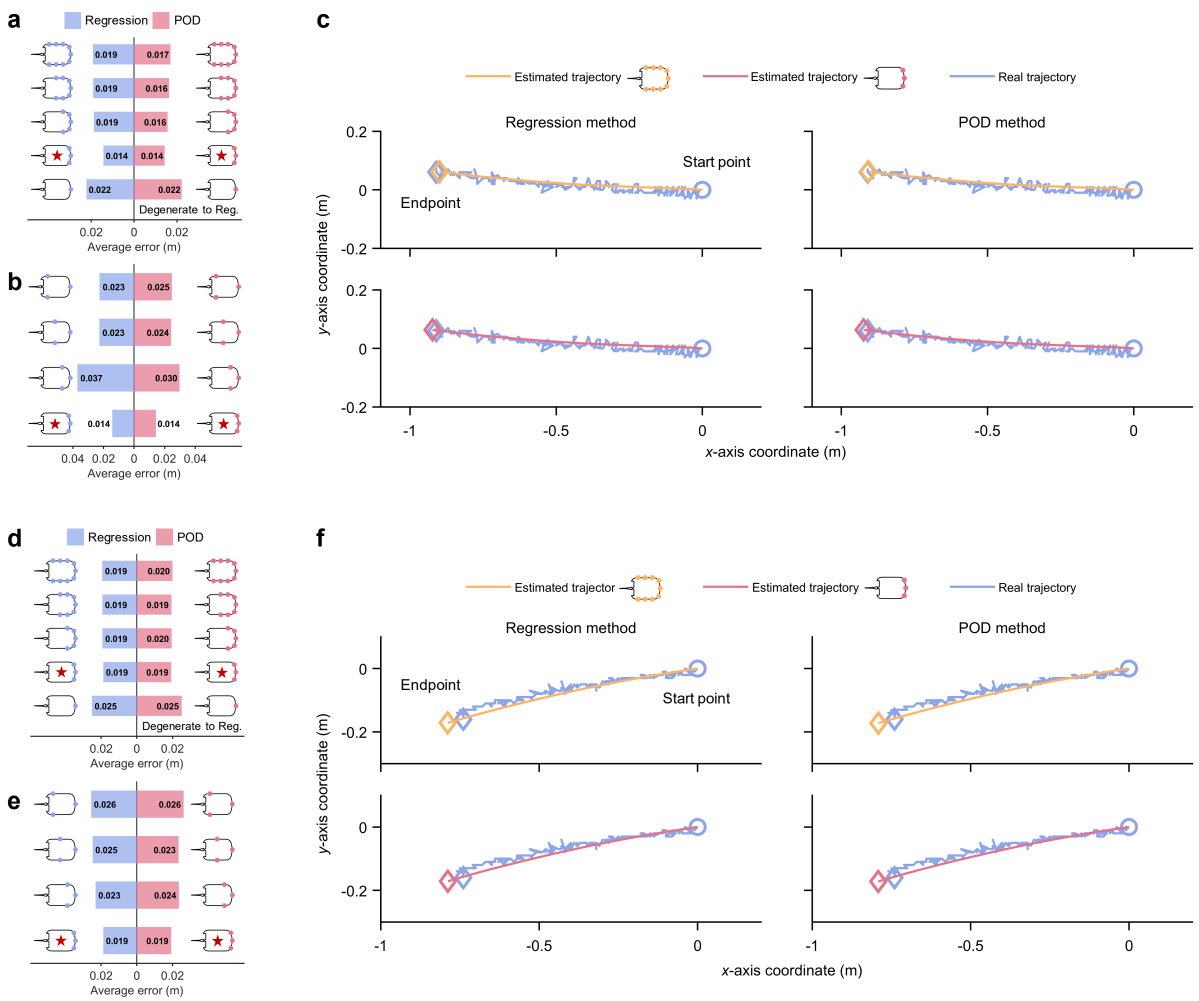

Supplementary Fig. 12 Trajectory estimation of more cases in the rectilinear motion. The points, lines, and colors in this figure follow the same legend as Fig. 4 and Fig. 6. The minimum error is reached by using L_1, O, R_1 for both methods. **a b c**, Frequency = 1.4 Hz, amplitude = 15°, and offset = 0°. **d e f**, Frequency = 1.8 Hz, amplitude = 10°, and offset = 0°.

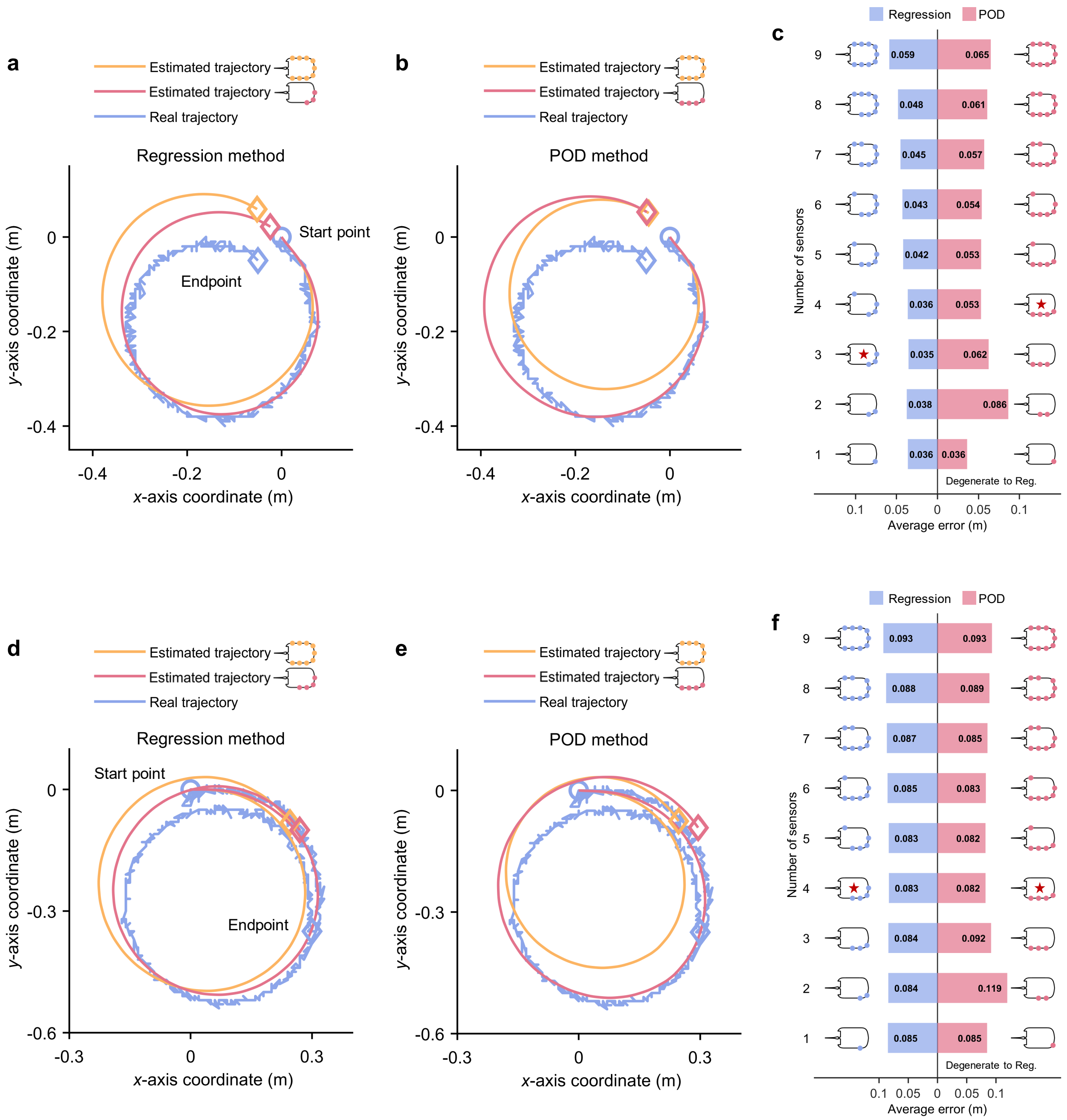

Supplementary Fig. 13 Trajectory estimation of more cases in the turning motion. The points, lines, and colors in this figure follow the same legend as Fig. 5 and Fig. 6. The minimum error is reached by using three or four sensors on the right front part for both methods. **a b c**, Frequency = 1.1 Hz, amplitude = 20°, and offset = 30°. **d e f**, Frequency = 1.3 Hz, amplitude = 20°, and offset = 25°.

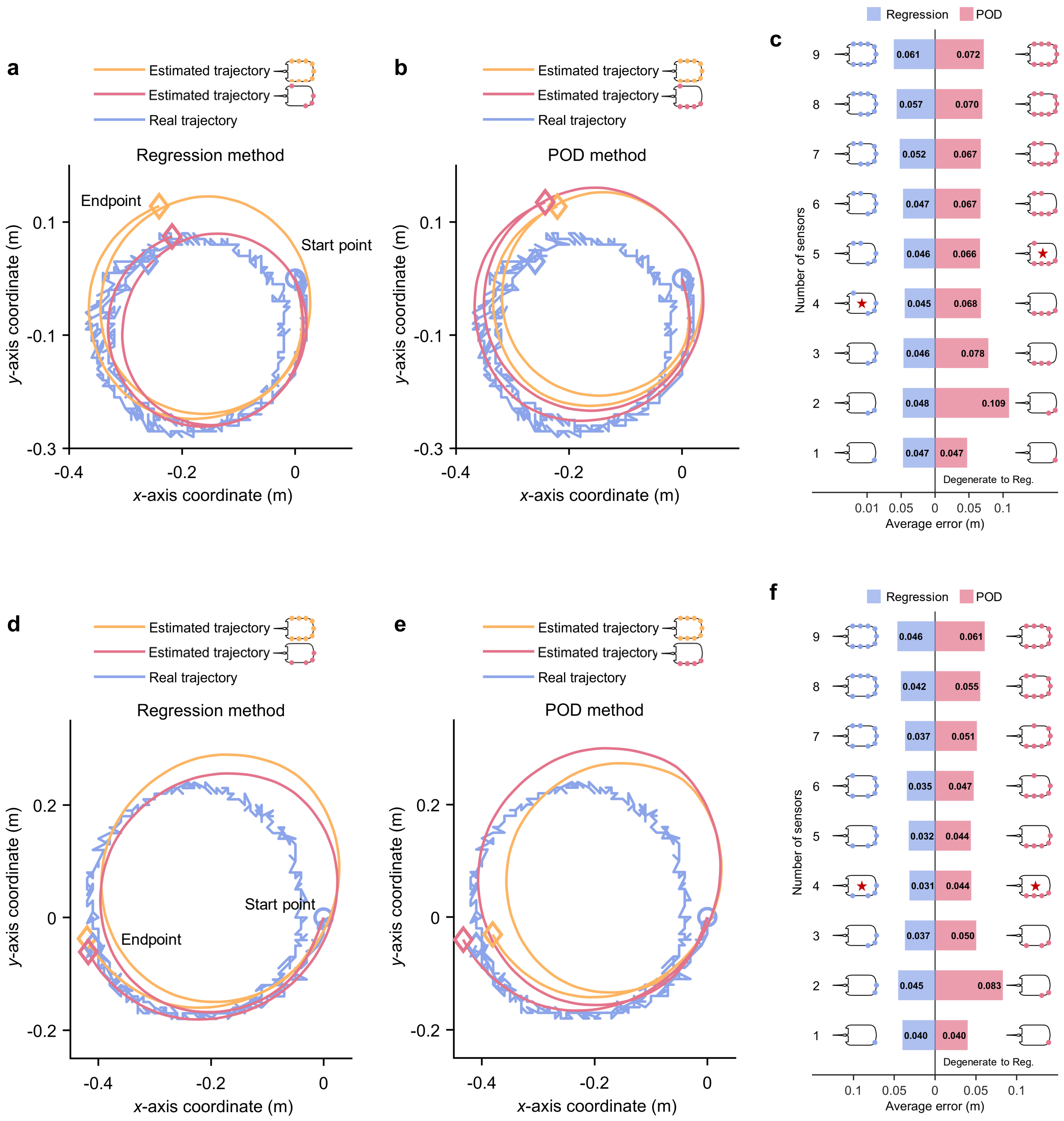

Supplementary Fig. 14 Trajectory estimation of more cases in the turning motion. The points, lines, and colors in this figure follow the same legend as Fig. 5 and Fig. 6. The minimum error is reached by using three or four sensors on the right front part for both methods. **a b c**, Frequency = 1.5 Hz, amplitude = 20°, and offset = 35°. **d e f**, Frequency = 1.7 Hz, amplitude = 20°, and offset = 30°.

Trajectory estimation of more cases under varying oscillation parameters

The decomposition and trajectory estimation results for rectilinear and turning motions under varying oscillation parameters are shown in Supplementary Figs. 15-18 and Supplementary Movies 5-8. Overall, the pressure variations can be decomposed as described in the manuscript. And the optimal combinations of sensors for the trajectory estimation are consistent with our conclusions in the manuscript.

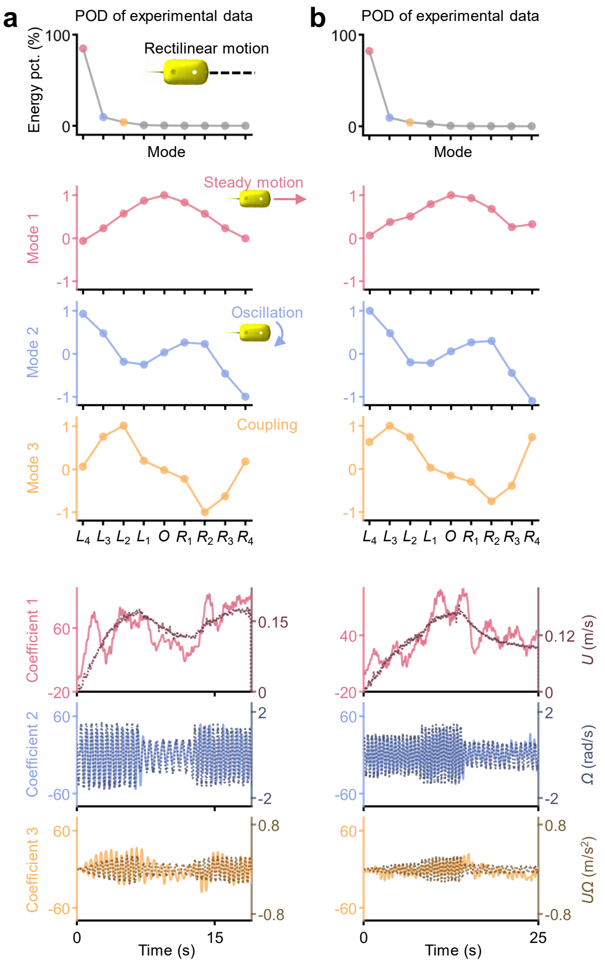

Supplementary Fig. 15 Mode decomposition (POD) results of the experimental hydrodynamic pressure data under varying oscillation parameters in the rectilinear motion. The points, lines, and colors in this figure follow the same legend as Fig. 2. The first three modes almost occupy all the energy. Mode 1 and coefficient 1 are related to the steady motion with varying forward velocity. Mode 2 and coefficient 2 are related to the oscillation with varying amplitude and frequency. Mode 3 and coefficient 3 are related to other coupling motions. **a**, The oscillation parameters are frequency = 1.8 Hz, amplitude = 30°, offset = 0°, then frequency = 1.4 Hz, amplitude = 20°, offset = 0°, and finally frequency = 2 Hz, amplitude = 25°, offset = 0°. **b**, The oscillation parameters are frequency = 1.8 Hz, amplitude = 20°, offset = 0°, then frequency = 2 Hz, amplitude = 25°, offset = 0°, and finally frequency = 1.5 Hz, amplitude = 15°, offset = 0°.

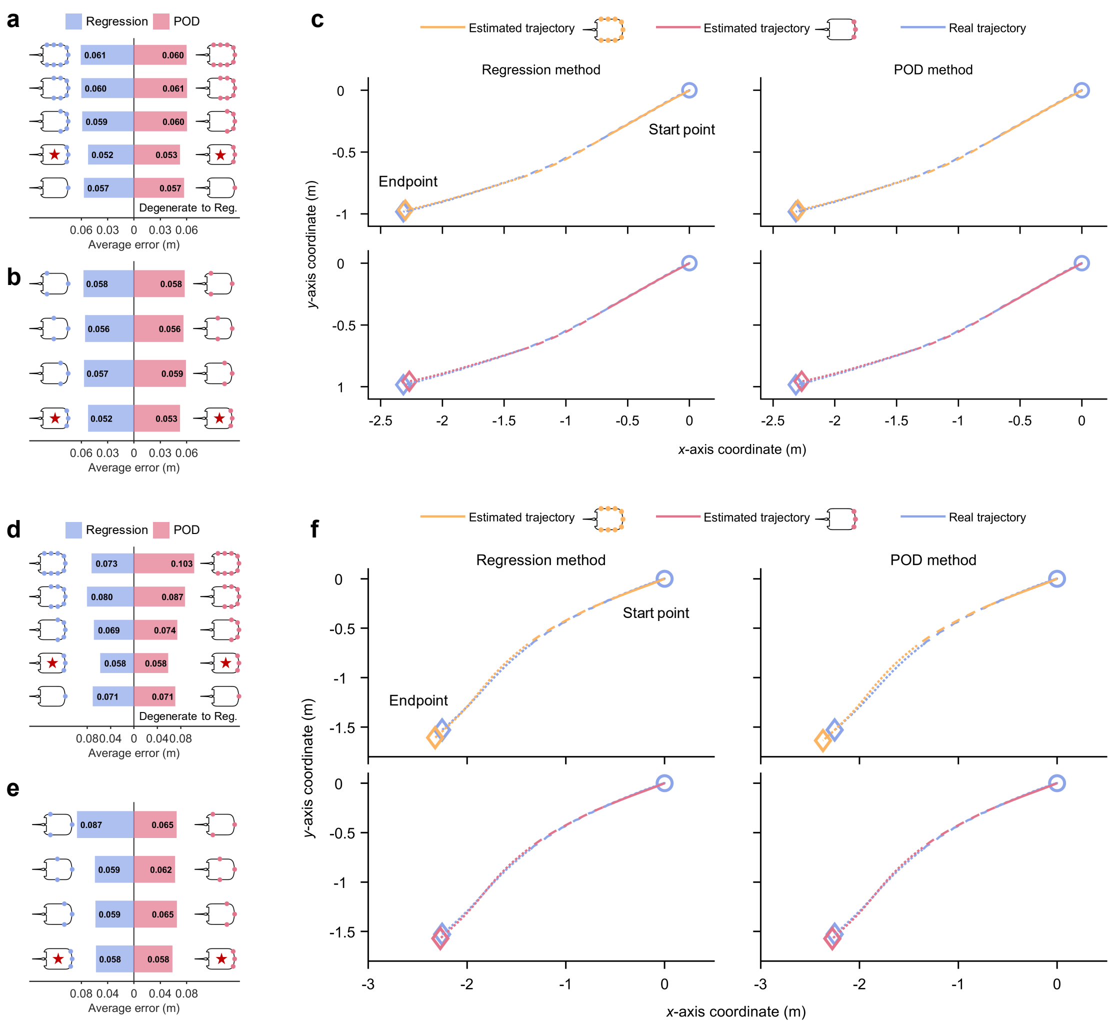

Supplementary Fig. 16 The trajectory estimation can be improved by using the optimal combination of pressure sensors, compared with using all nine sensors under varying oscillation parameters in the rectilinear motion. The points, lines, and colors in this figure follow the same legend as Fig. 4 and Fig. 6. **a b c**, The oscillation parameters are frequency = 1.8 Hz, amplitude = 30°, offset = 0°, then frequency = 1.4 Hz, amplitude = 20°, offset = 0°, and finally frequency = 2 Hz, amplitude = 25°, offset = 0° (detailed in Supplementary Movie 5). **d e f**, The oscillation parameters are frequency = 1.8 Hz, amplitude = 20°, offset = 0°, then frequency = 2 Hz, amplitude = 25°, offset = 0°, and finally frequency = 1.5 Hz, amplitude = 15°, offset = 0° (detailed in Supplementary Movie 6).

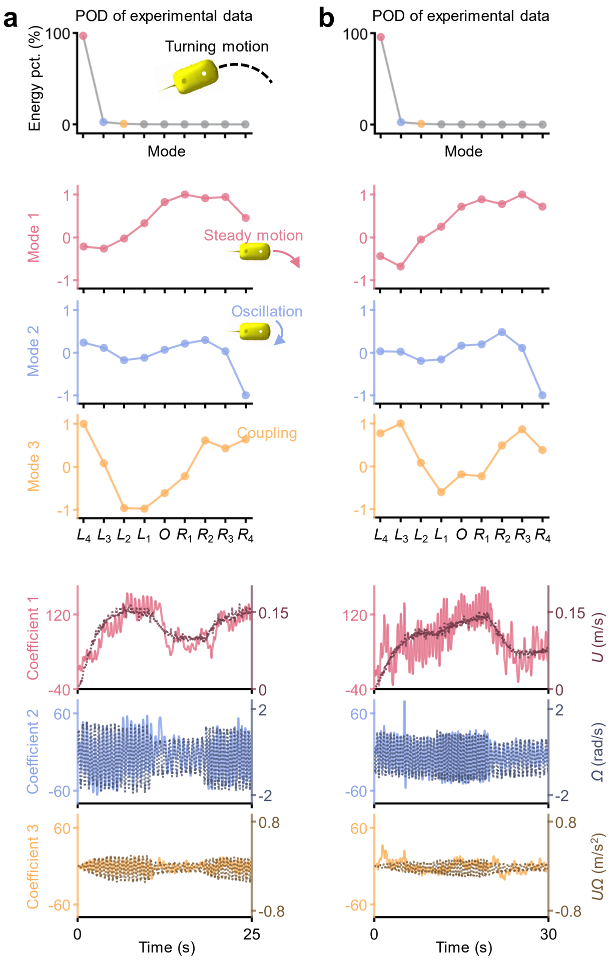

Supplementary Fig. 17 Mode decomposition (POD) results of the experimental hydrodynamic pressure data under varying oscillation parameters in the turning motion. The points, lines, and colors in this figure follow the same legend as Fig. 3. The first three modes almost occupy all the energy. Mode 1 and coefficient 1 are related to the steady motion with varying forward velocity. Mode 2 and coefficient 2 are related to the oscillation with varying amplitude and frequency. Mode 3 and coefficient 3 are related to other coupling motions. **a**, The oscillation parameters are frequency = 1.8 Hz, amplitude = 30°, offset = 20°, then frequency = 1.5 Hz, amplitude = 20°, offset = 20°, and finally frequency = 2 Hz, amplitude = 25°, offset = 20°. **b**, The oscillation parameters are frequency = 1.8 Hz, amplitude = 20°, offset = 25°, then frequency = 2 Hz, amplitude = 20°, offset = 20°, and finally frequency = 1.4 Hz, amplitude = 20°, offset = 30°.

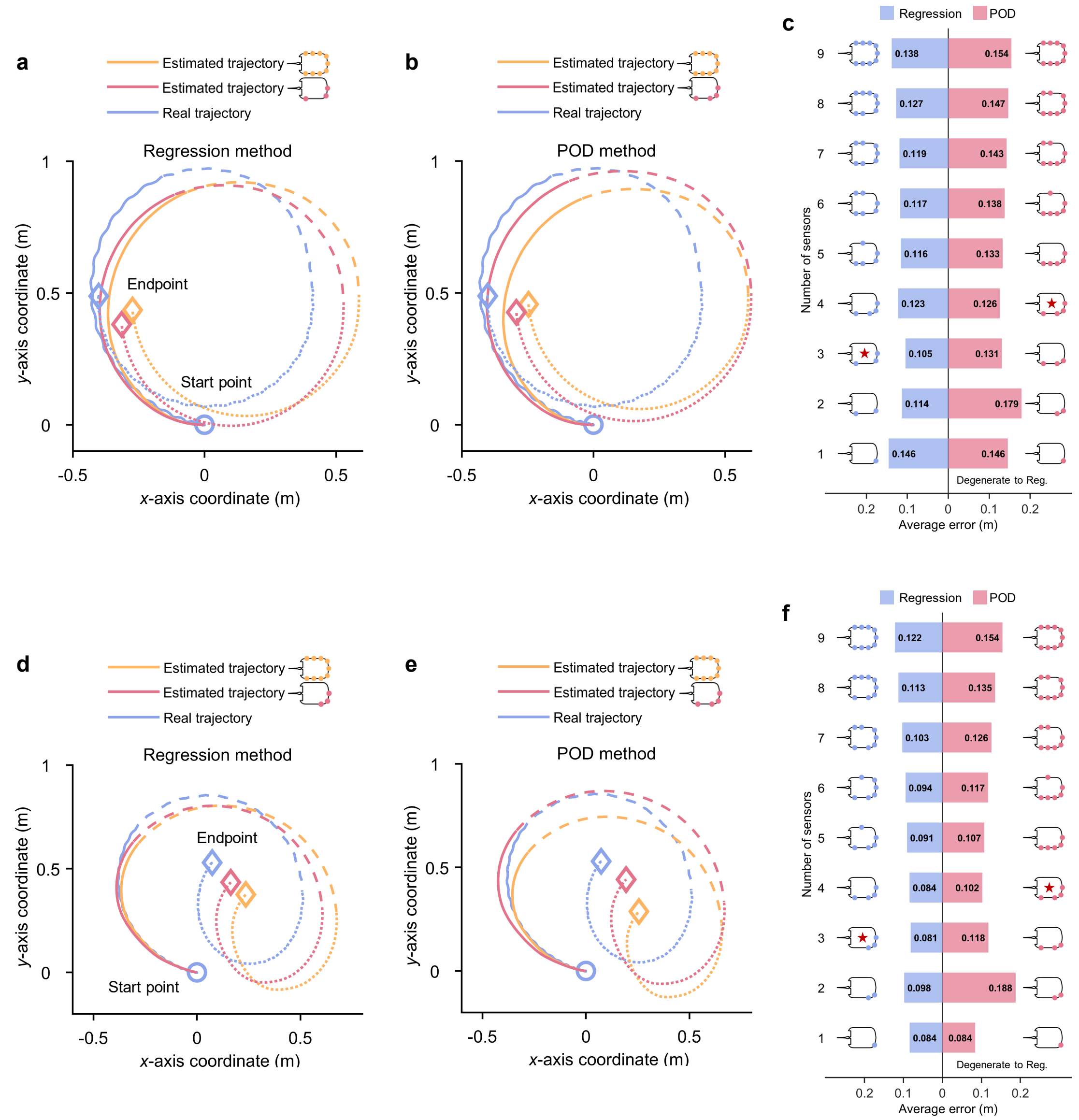

Supplementary Fig. 18 The trajectory estimation can be improved by using the optimal combination of pressure sensors, compared with using all nine sensors under varying oscillation parameters in the turning motion. The points, lines, and colors in this figure follow the same legend as Fig. 5 and Fig. 6. **a b c**, The oscillation parameters are frequency = 1.8 Hz, amplitude = 30°, offset = 20°, then frequency = 1.5 Hz, amplitude = 20°, offset = 20°, and finally frequency = 2 Hz, amplitude = 25°, offset = 20° (detailed in Supplementary Movie 7). **d e f**, The oscillation parameters are frequency = 1.8 Hz, amplitude = 20°, offset = 25°, then frequency = 2 Hz, amplitude = 20°, offset = 20°, and finally frequency = 1.4 Hz, amplitude = 20°, offset = 30° (detailed in Supplementary Movie 8).

Supplementary Note 9

Hydrogen bubble flow visualization

Due to the difficulty of visualizing the flow structures around a free-swimming fish-like robot, we fix the fish body in the water channel for an approximate simulation (Supplementary Fig. 19). The fish body is driven by a motor. The velocity of the uniform flow is set to be the same as the swimming velocity. The oscillation angle of the fish body is also the same as that in free swimming. The visualization results are shown in Supplementary Movies 2 to 4. Due to the discrete output of the motor and the oscillation of the mechanical structure, there exist some small sudden changes during the oscillation of the fish body. The real-time oscillation angles are captured from the movies, which are shown in Supplementary Fig. 20. The oscillations of the fish body follow the same sinusoidal functions as free-swimming overall. In the rectilinear motion, the amplitude, frequency, and offset of the oscillation are 7°, 1.7 Hz, and 0°, respectively. In the turning motion, the amplitude, frequency, and offset of the oscillation are 5.5°, 1.9 Hz, and 9°, respectively.

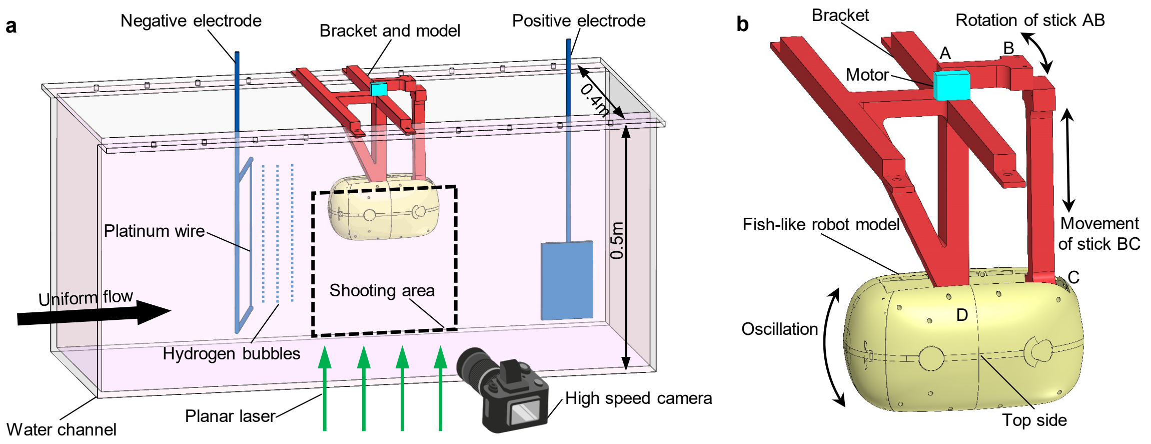

Supplementary Fig. 19 Experimental platforms for flow visualization enabled by the hydrogen bubble technique. **a**, The experiments are carried out in a low-turbulence water channel with a fish-like robot model and a bracket. The positive and negative electrodes for water electrolysis are placed in water. Hydrogen bubbles are generated at a specific frequency from the platinum wire of the negative electrode. Hydrogen bubble filaments move forward with the flow and then deform under the impact of the moving model, visualizing the complicated flow structures. The

images are enhanced by a planer laser and recorded by a high-speed camera from the top side of the model. **b**, Details about the fish-like robot model and the bracket. A motor is used to drive the model according to a sinusoidal function. The motor drives stick AB to rotate, and the motion is transmitted to the oscillation of the model through stick BC.

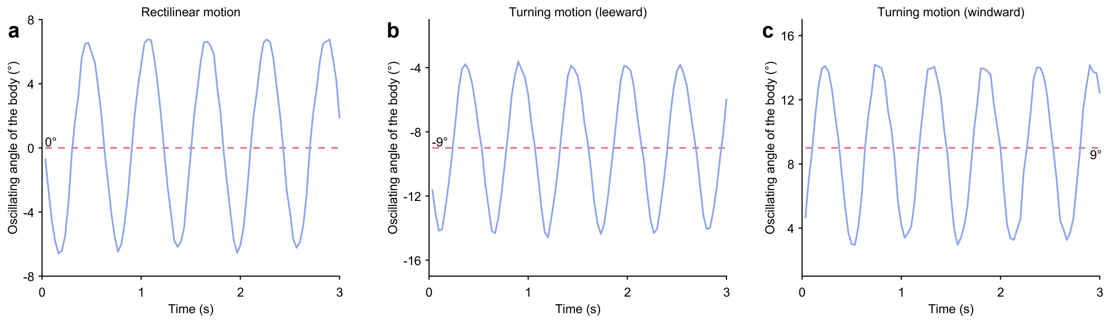

Supplementary Fig. 20 The oscillation angles of the fish body in flow visualization. **a**, Rectilinear motion. **b**, Leeward in the turning motion. **c**, Windward in the turning motion. Blue lines represent the oscillation angles. Red lines represent the offset values.

Supplementary Note 10

Computational fluid dynamics (CFD) simulation based on IBAMR

We simulate the unsteady flow fields surrounding a 3D body using the Immersed Boundary (IB) method. In the IB formulation for fluid-structure interaction (FSI), the fluid is represented using an Eulerian framework, while the structure is modeled with a Lagrangian approach. The coupling between the Eulerian and Lagrangian variables is described through integral equations with delta function kernels. We conduct CFD simulations using the open-source software IBAMR (https://ibamr.github.io/). This tool provides a distributed-memory parallel implementation of the IB method, incorporating the Cartesian grid adaptive mesh refinement (AMR) technique[5]. The Robin boundary conditions implemented using the Structured Adaptive Mesh Refinement Application Infrastructure (SAMRAI) library have been used to generate the appropriate boundary of the computational domain.

Based on the formulation outlined[6], the corresponding IB equations are presented as follows:

$$\rho \frac{\mathrm{D}\mathbf{u}}{\mathrm{D}t}(\mathbf{x},t) = -\nabla p(\mathbf{x},t) + \mu \nabla^2 \mathbf{u}(\mathbf{x},t) + \mathbf{f}(\mathbf{x},t) \tag{S6}$$

$$\nabla \cdot \mathbf{u}(\mathbf{x},t) = 0 \tag{S7}$$

$$\mathbf{U}_f(\mathbf{s},t) = \int_{\Omega} \mathbf{u}(\mathbf{x},t)\delta[\mathbf{x} - \chi(\mathbf{s},t)]\mathrm{d}\mathbf{x} \tag{S8}$$

$$\mathbf{U}(\mathbf{s},t) = \mathbf{V}(\mathbf{s},t) \tag{S9}$$

$$\frac{\partial \chi}{\partial t}(\mathbf{s},t) = \mathbf{V}(\mathbf{s},t) \tag{S10}$$

$$\mathbf{f}(\mathbf{x},t) = \int_{\Omega_c} F(\mathbf{s},t)\delta[\mathbf{x} - \chi(\mathbf{s},t)]\mathrm{d}\mathbf{x} \tag{S11}$$

Here, ρ denotes the fluid density; $\mathrm{D}/\mathrm{D}t = \partial/\partial t + \mathbf{u}(\mathbf{x},t) \cdot \nabla$ represents the convective derivative; $\mathbf{u}(\mathbf{x},t)$ is the fluid velocity; $\mathbf{x}$ refers to the spatial coordinates. The static pressure is given by $p(\mathbf{x},t)$, and μ represents the fluid viscosity. The term $\mathbf{f}(\mathbf{x},t)$ is the body force responsible for enforcing the constraints on

the body. $\mathbf{U}_f(\mathbf{s},t)$ is the Lagrangian velocity of the body in simulation. $\mathbf{V}(\mathbf{s},t)$ is the prescribed velocity. The position of a material point $\mathbf{s}$ at time t is represented by $\chi(\mathbf{s},t)$, and δ is the Dirac delta function. The domain Ω refers to the physical space, while Ω_c corresponds to the regions of the body where constraints on its motion are applied. The Lagrangian velocity of the solid body can be defined as an explicit function of time:

$$\left(\mathbf{U}_f\right)_{l,m,n} = \mathbf{U}_r + \mathbf{W}_r \times \mathbf{R}_{l,m,n} \tag{S12}$$

where $\mathbf{U}_r$ and $\mathbf{W}_r$ refer to the linear and angular velocities of the center of mass (COM) of the body in simulation, respectively. $\mathbf{R}_{l,m,n}$ is the radius vector from the COM to the physical position $\mathbf{x}_{l,m,n}$ of curvilinear mesh node (l,m,n). The positions of the constrained parts of the robot are corrected by

$$\mathbf{x}_{l,m,n}^{n+1,k+1} = \mathbf{x}_{l,m,n}^{n} + \Delta t\, \mathbf{U}_f^{n+\frac{1}{2},k+1} \tag{S13}$$

where n is the time step number, k denotes the cycle number for iteration, and Δt is the time step size, respectively.

Simulation settings for the boxfish

To perform CFD analysis, we first create a total of 17,687 Lagrangian points according to the movement of the fish-like robot in the experiments, to describe the geometry of our fish-like robot, as shown in Supplementary Fig. 21**a**. The movement of the fish-like robot in CFD simulations are constrained to follow identical spatiotemporal trajectories in free-swimming experiments. The computational domain is modeled as a rectangular cuboid measuring 12.8 × 12.8 × 3.2 body length L. The boundary conditions are the symmetric boundary conditions. The time step of the simulation is set as $\Delta t = 0.0005s$. To obtain the hydrodynamic pressure variations, we set 2,415 probes distributed on the surface. The sampling frequency is 200Hz.

Simulation settings for the eel-like swimmer

The eel-like swimmer (Supplementary Fig. 21**b**), with a predetermined morphology, was constructed with a total of 17,481 points from elliptical disks based on specified axis width and height parameters, following the methodology outlined by Kern and Koumoutsakos[7] and Chao et al[8]. The swimmer's position relative to the flow is fixed, featuring in-place lateral oscillations. The numerical swimmer is characterized by a body length, denoted as L, and a maximal tailbeat amplitude, A. We maintain a constant dimensionless maximal tailbeat amplitude of $A = 0.1L$. The kinematics of the swimmer is defined as follows:

$$y(x,t) = \left(0.02 - 0.08\frac{x}{L} + 0.16\left(\frac{x}{L}\right)^2\right)\sin\left(2\pi\left(\frac{x}{L} - \frac{t}{T}\right)\right) \tag{S14}$$

Where $T = 1s$ denotes the period of the oscillation. In the simulations, the computational domain is modeled as a rectangular prism measuring $16 \times 8 \times 2L$. The boundary conditions include uniform oncoming flow with a velocity of 1.0 L/s on the left side, no-stress outflow boundary conditions on the right, and no-slip wall conditions on the remaining boundaries. The time step size is fixed at $0.001T$. To obtain the hydrodynamic pressure variations, we set 8,000 probes distributed on the surface. The sampling frequency is 100Hz.

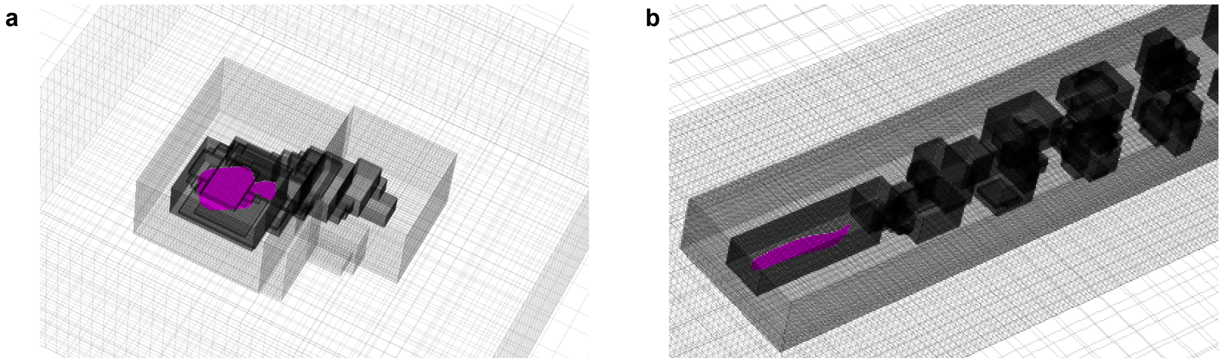

Supplementary Fig. 21 The CFD simulation environments and varying mesh densities. **A**, Boxfish model. **b**, Eel-like model.

Supplementary Note 11

Generalizability verification by self-velocity estimation in complex flows with vortices

Another set of results for the self-velocity estimation of the focal fish-like robot is shown in Supplementary Fig. 22. The phenomenon is consistent with the description in the main text. The POD method is more robust than the regression method in most cases. When the longitudinal distance is 5 to 30 cm, the sensors of the focal fish-like robot are affected by the vortices from the leading fish-like robot. In these situations, the POD method can estimate the self-velocity more accurately than the regression method. When the longitudinal distance is larger than 30 cm, the influence of the vortices on the focal fish-like robot is insignificant, and the estimation errors of both methods decrease.

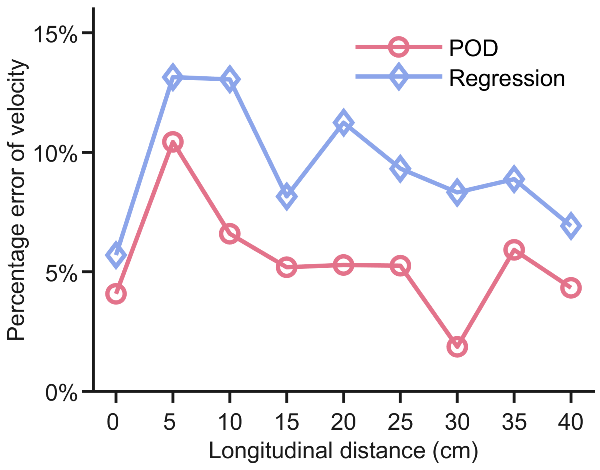

Supplementary Fig. 22 More results for errors of the estimated velocity for the focal fish-like robot in complex flows with vortices. The error is quantified as a percentage relative to the forward velocity. Red lines and circles represent the average errors by the POD method. Blue lines and diamonds represent the average errors by the regression method.

Model of error analysis

To compare the impact of the sensor error on the estimated velocity of the two methods in complex flows with vortices, we attempt to establish a rough model. For the regression method, the estimated velocity is calculated by

$$\widetilde{U}_{\text{reg}} = \min_{U} \|\mathbf{p} - (\mathbf{a}U^2 + \mathbf{b}U + \mathbf{c})\|^2 \tag{S15}$$

where $\mathbf{p} \in \mathbb{R}^N$ represents the average value of the pressure data. $\mathbf{a}$, $\mathbf{b}$, $\mathbf{c} \in \mathbb{R}^N$ represent the regression coefficients. Here, N is equal to nine. The estimated velocity $\widetilde{U}_{\text{reg}}$ satisfies the first-order condition

$$\sum_{i=1}^{9} \left[p_i - \left(a_i \widetilde{U}_{\text{reg}}^2 + b_i \widetilde{U}_{\text{reg}} + c_i\right)\right] \cdot \left(2a_i \widetilde{U}_{\text{reg}} + b_i\right) = 0 \tag{S16}$$

where p_i, a_i, b_i, c_i represent the components of $\mathbf{p}$, $\mathbf{a}$, $\mathbf{b}$, $\mathbf{c}$. Taking the derivative with respect to p_k,

$$\begin{aligned} 0 = &\sum_{i=1}^{9} \frac{\partial}{\partial p_k} p_i \cdot \left(2a_i \widetilde{U}_{\text{reg}} + b_i\right) + \sum_{i=1}^{9} p_i \cdot \frac{\partial}{\partial p_k} \left(2a_i \widetilde{U}_{\text{reg}} + b_i\right) \\ &- \sum_{i=1}^{9} \frac{\partial}{\partial p_k} \left(a_i \widetilde{U}_{\text{reg}}^2 + b_i \widetilde{U}_{\text{reg}} + c_i\right) \cdot \left(2a_i \widetilde{U}_{\text{reg}} + b_i\right) \\ &- \sum_{i=1}^{9} \left(a_i \widetilde{U}_{\text{reg}}^2 + b_i \widetilde{U}_{\text{reg}} + c_i\right) \cdot \frac{\partial}{\partial p_k} \left(2a_i \widetilde{U}_{\text{reg}} + b_i\right) \end{aligned} \tag{S17}$$

Considering that p_i is independent of each other, so

$$\begin{aligned} \frac{\partial \widetilde{U}_{\text{reg}}}{\partial p_k} &= \frac{-2a_k \widetilde{U}_{\text{reg}} - b_k}{\sum_{i=1}^{9}(2a_i p_i) - \sum_{i=1}^{9}\left(6{a_i}^2 \widetilde{U}_{\text{reg}}^2 + 6a_i b_i \widetilde{U}_{\text{reg}} + {b_i}^2 + 2a_i c_i\right)} \\ &= \frac{-2a_k \widetilde{U}_{\text{reg}} - b_k}{2\mathbf{a} \cdot \mathbf{p} - \left(6\|\mathbf{a}\|^2 \widetilde{U}_{\text{reg}}^2 + 6\mathbf{a} \cdot \mathbf{b} \widetilde{U}_{\text{reg}} + \|\mathbf{b}\|^2 + 2\mathbf{a} \cdot \mathbf{c}\right)} \end{aligned} \tag{S18}$$

For the POD method, the estimated velocity is calculated by

$$\widetilde{U}_{\text{pod}} = \min_{U} |\text{coef}_1 - (aU^2 + bU + c)|^2 \tag{S19}$$

where coef_1 represents the average value of the coefficient of mode 1 in POD. a, b, c represent the regression coefficients between coef_1 and the velocity. The estimated velocity $\widetilde{U}_{\text{pod}}$ satisfies the first-order condition

$$\left[\text{coef}_1 - \left(a \widetilde{U}_{\text{pod}}^2 + b \widetilde{U}_{\text{pod}} + c\right)\right] \cdot \left(2a \widetilde{U}_{\text{pod}} + b\right) = 0 \tag{S20}$$

Taking the derivative with respect to coef_1, it is obtained that

$$\frac{\partial \widetilde{U}_{\mathrm{pod}}}{\partial \mathrm{coef}_1} = \frac{-2a\widetilde{U}_{\mathrm{pod}} - b}{2a\,\mathrm{coef}_1 - \left(6a^2\widetilde{U}_{\mathrm{pod}}^2 + 6ab\widetilde{U}_{\mathrm{pod}} + b^2 + 2ac\right)} \tag{S21}$$

Considering that $\mathrm{coef}_1 = \mathbf{p} \cdot \mathbf{u^1}$ in POD where $\mathbf{u^1}$ represents mode 1, the derivative of $\widetilde{U}_{\mathrm{pod}}$ with respect to p_k is expressed as

$$\begin{aligned}\frac{\partial \widetilde{U}_{\mathrm{pod}}}{\partial p_k} &= \frac{\partial \widetilde{U}_{\mathrm{pod}}}{\partial \mathrm{coef}_1}\frac{\partial \mathrm{coef}_1}{\partial p_k} \\ &= \frac{\left(-2a\widetilde{U}_{\mathrm{pod}} - b\right)\frac{\partial \mathrm{coef}_1}{\partial p_k}}{\sum_{i=1}^{9} 2au_i^1 p_i - \left(6a^2\widetilde{U}_{\mathrm{pod}}^2 + 6ab\widetilde{U}_{\mathrm{pod}} + b^2 + 2ac\right)} \\ &= \frac{\left(-2au_k^1\widetilde{U}_{\mathrm{pod}} - bu_k^1\right)\frac{\partial \mathrm{coef}_1}{\partial p_k}\frac{1}{u_k^1}}{2a\mathbf{u^1} \cdot \mathbf{p} - \left(6a^2\widetilde{U}_{\mathrm{pod}}^2 + 6ab\widetilde{U}_{\mathrm{pod}} + b^2 + 2ac\right)}\end{aligned} \tag{S22}$$

where u_k^1 represents the component of mode 1. Comparing Eq. (S18) and Eq. (S22), the derivative of estimated velocity with respect to p_k have similar forms. Here, we only analyze the effect of errors in the average value of the pressure data. In the regression method, fluctuating errors can be eliminated by taking the average value to some extent. In the POD method, only the mode corresponding to the steady-state motion is extracted and used for estimation. So errors in the average value have a greater impact on the estimation results. As shown in Supplementary Fig. 23, the coefficients in two methods $\mathbf{a}$ and $a\mathbf{u^1}$, $\mathbf{b}$ and $b\mathbf{u^1}$, $\mathbf{c}$ and $c\mathbf{u^1}$ have similar shapes and values. So the term $\frac{\partial \mathrm{coef}_1}{\partial p_k}\frac{1}{u_k^1}$ determines the difference between $\frac{\partial \widetilde{U}_{\mathrm{pod}}}{\partial p_k}$ and $\frac{\partial \widetilde{U}_{\mathrm{reg}}}{\partial p_k}$. According to the POD algorithm, the modes are dependent on $\mathbf{p}$

$$\mathrm{coef}_1 = \mathbf{p} \cdot \mathbf{u^1} = \sum_{i=1}^{9} p_i u_i^1(\mathbf{p}) \tag{S23}$$

So

$$\frac{\partial \mathrm{coef}_1}{\partial p_k}\frac{1}{u_k^1} = 1 + \frac{p_k}{u_k^1}\frac{\partial u_k^1}{\partial p_k} + \sum_{i \neq k} \frac{p_i}{u_k^1}\frac{\partial u_i^1}{\partial p_k} \tag{S24}$$

where p_k and u_k^1 have the same signs. The effect of p_k on u_k^1 is positive because the increase of p_k will raise the component proportion of u_k^1 in the vector $\mathbf{u^1}$. In contrast, the effect of p_k on $u_i^1 (i \neq k)$ is negative because the increase of p_k will decrease the component proportion of pressure data of other locations. In our experiments, the absolute value of sensors located in the vortex flow area, which are easily affected, is insignificant. So the term $\frac{p_k}{u_k^1}\frac{\partial u_k^1}{\partial p_k}$ is positive but small. The negative term $\sum_{i \neq k} \frac{p_i}{u_k^1}\frac{\partial u_i^1}{\partial p_k}$ can compensate for the error, causing that the final value of Eq. (S24) is smaller than one and then the estimation error of POD method is smaller than the regression method. To sum up, the POD method can extract the main components and reduce the impact of sensor errors on results, especially the sensors that are not important in the system.

It should be clarified that the proof here is only a rough result and is not strict enough. We hope that it can help to explain the better performance of the POD method than the regression method in the velocity estimation in complex flows with vortices. However, it cannot explain why the performance of the regression method is slightly better in the trajectory estimation of a free-swimming fish-like robot. The reason may be that the values of the terms in Eq. (S24) are very close when the interference is insignificant.

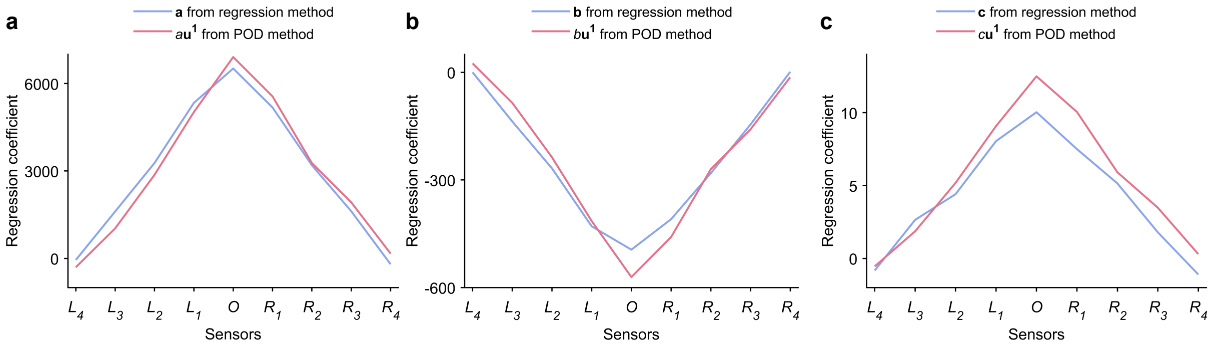

Supplementary Fig. 23 Regression coefficients of the regression method and the POD method. **a**, Coefficients of the quadratic term. **b**, Coefficients of the linear term. **c**, Coefficients of the constant term. Red lines and blue lines represent the POD method and the regression method respectively.

Supplementary Note 12

POD for velocity field around the fish-like robot

We conduct a preliminary POD analysis of the velocity field around a swimming fish-like robot in a 3D CFD simulation. It can be seen in Supplementary Fig. 24 that the velocity field can also be decomposed by POD. Mode 1 represents the swimming velocity forward with an approximately constant value on the surface and coefficient 1 can reflect the acceleration of the robot. Mode 2 and coefficient 2 reflect the oscillation velocity. This demonstrates that POD method can be applied not only to analyze pressure field data but also to analyze velocity field data. In the future, we intend to perform an in-depth exploration of the application of POD to both the velocity and pressure fields around fish-like robots or real fish, aiming to extract more valuable information for underwater perception. This will align with the capability of real lateral lines to simultaneously perceive both flow velocity and pressure signals.

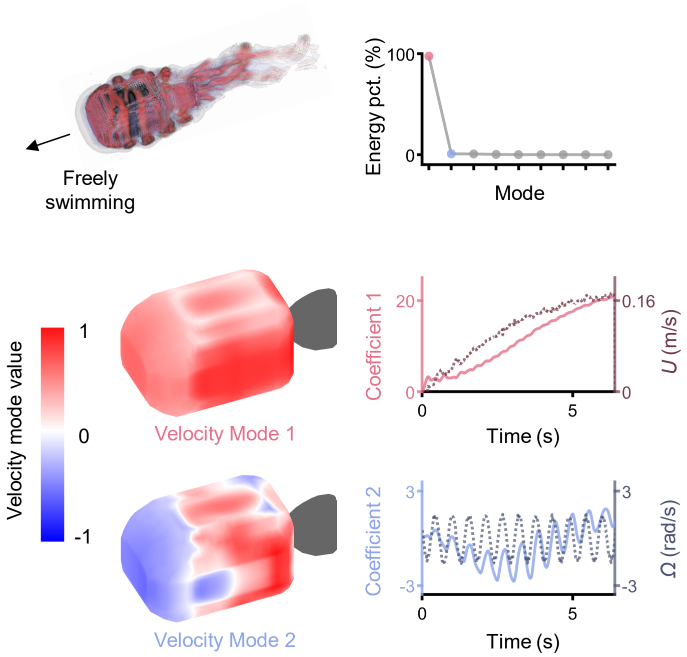

Supplementary Fig. 24 Mode decomposition (POD) of the three-dimensional velocity field value from CFD simulations for numerical boxfish, including the flow field visualized by isosurfaces of the Q-criterion, energy proportions, modes and coefficients. The boxfish model follows the

same kinematics as the experiment with oscillation frequency = 1.8 Hz, amplitude = 30°, offset = 0°.

References

[1] Berkooz, G., Holmes, P. & Lumley, J. L. The proper orthogonal decomposition in the analysis of turbulent flows. *Annual review of fluid mechanics* **25**, 539–575 (1993).

[2] Anderson, J. *EBOOK: Fundamentals of Aerodynamics (SI units)* (McGraw hill 2011).

[3] Lighthill, S. J. Estimates of pressure differences across the head of a swimming clupeid fish. *Philosophical Transactions of the Royal Society of London. Series B: Biological Sciences* **341**, 129-140 (1993).

[4] Zheng, X., Wang, W., Xiong, M., & Xie, G. Online state estimation of a fin-actuated underwater robot using artificial lateral line system. *IEEE Transactions on Robotics* **36**, 472-487 (2020).

[5] Chao, L. M., Jia, L. & Li, L. Tailbeat perturbations improve swimming efficiency in self-propelled flapping foils. *Journal of Fluid Mechanics* **984**, A46 (2024).

[6] Griffith, B. E. & Patankar, N. A. Immersed methods for fluid-structure interaction. *Annual review of fluid mechanics* **52**, 421-448 (2020).

[7] Kern, S. & Koumoutsakos, P. Simulations of optimized anguilliform swimming. *Journal of Experimental Biology* **209**, 4841-4857 (2006).

[8] Chao, L. M., Couzin, I. D. & Li, L. On turning maneuverability in self-propelled burst-and-coast swimming. *Physics of Fluids* **36** (2024).
