# Supplementary material for: An interpretable approach to estimate the self-motion in fish-like robots using mode decomposition analysis
Source: Nat Commun. 2025 Apr 24;16:3887. doi: 10.1038/s41467-025-58880-6 (PMC12022055; doi:10.1038/s41467-025-58880-6)
Supplement: Supplementary file 2 — Description of Additional Supplementary Files [file 41467_2025_58880_MOESM2_ESM.pdf]

File Name: Supplementary Movie 1
Description: Experiments of one free-swimming fish-like robot

File Name: Supplementary Movie 2
Description: Flow visualization in rectilinear motion

File Name: Supplementary Movie 3
Description: Flow visualization in turning motion (leeward)

File Name: Supplementary Movie 4
Description: Flow visualization in turning motion (windward)

File Name: Supplementary Movie 5
Description: Rectilinear motion under varying oscillation parameters Case I

File Name: Supplementary Movie 6
Description: Rectilinear motion under varying oscillation parameters Case II

File Name: Supplementary Movie 7
Description: Turning motion under varying oscillation parameters Case I

File Name: Supplementary Movie 8
Description: Turning motion under varying oscillation parameters Case II

File Name: Supplementary Movie 9
Description: Experiments of two towed fishlike robots swimming in a tank
